# Category systems for real-world scenes

**DOI:** 10.1167/jov.21.2.8

**Published:** 2021-02-17

**Authors:** Matt D. Anderson, Erich W. Graf, James H. Elder, Krista A. Ehinger, Wendy J. Adams

**Affiliations:** 1 Centre for Vision and Cognition, Psychology, University of Southampton, Southampton, UK; 2 Centre for Vision and Cognition, Psychology, University of Southampton, Southampton, UK; 3 Centre for Vision Research, Department of Psychology, Department of Electrical Engineering and Computer Science, York University, Toronto, Ontario, Canada; 4 School of Computing and Information Systems, The University of Melbourne, Melbourne, Australia; 5 Centre for Vision and Cognition, Psychology, University of Southampton, Southampton, UK

**Keywords:** high-level scene perception, scene categorization, clustering

## Abstract

Categorization performance is a popular metric of scene recognition and understanding in behavioral and computational research. However, categorical constructs and their labels can be somewhat arbitrary. Derived from exhaustive vocabularies of place names (e.g., Deng et al., 2009), or the judgements of small groups of researchers (e.g., Fei-Fei, Iyer, Koch, & Perona, 2007), these categories may not correspond with human-preferred taxonomies. Here, we propose clustering by increasing the rand index via coordinate ascent (CIRCA): an unsupervised, data-driven clustering method for deriving ground-truth scene categories. In Experiment 1, human participants organized 80 stereoscopic images of outdoor scenes from the Southampton-York Natural Scenes (SYNS) dataset (Adams et al., 2016) into discrete categories. In separate tasks, images were grouped according to i) semantic content, ii) three-dimensional spatial structure, or iii) two-dimensional image appearance. Participants provided text labels for each group. Using the CIRCA method, we determined the most representative category structure and then derived category labels for each task/dimension. In Experiment 2, we found that these categories generalized well to a larger set of SYNS images, and new observers. In Experiment 3, we tested the relationship between our category systems and the spatial envelope model (Oliva & Torralba, 2001). Finally, in Experiment 4, we validated CIRCA on a larger, independent dataset of same-different category judgements. The derived category systems outperformed the SUN taxonomy (Xiao, Hays, Ehinger, Oliva, & Torralba, 2010) and an alternative clustering method (Greene, 2019). In summary, we believe this novel categorization method can be applied to a wide range of datasets to derive optimal categorical groupings and labels from psychophysical judgements of stimulus similarity.

## Category systems for real-world scenes

The visual properties of real-world environments have enormous heterogeneity: no two scenes are exactly alike. Scene categories allow us to organize environments into meaningful, discrete classes that represent their statistical regularities, and provide a coarse, efficient description of the environment. Category membership provides information about the probable activities, objects, and layouts associated with a scene, and serves as a convenient descriptor—most people can easily visualize the typical characteristics of forests or beaches, for example. It is unsurprising, then, that categorization performance is a popular metric of scene understanding in behavioral and computational research.

Scene categorization is achieved with impressive efficiency and minimal cognitive resources: novel images can be categorized from brief presentation durations (Fei-Fei et al., 2007; Potter, 1976), from only foveal, or only peripheral, visual information (Larson, Freeman, Ringer, & Loschky, 2014; Larson & Loschky, 2009), and in the near-absence of attention (Li, VanRullen, Koch, & Perona, 2002). The computational processes that underpin this ability have been extensively investigated (Malcolm, Groen, & Baker, 2016); however, most research does not scrutinize the taxonomical structure of applied category systems, that is, the ontological “realness” of the individual categories, the lawfulness of the categorical boundaries, the number of categories, and so on. In this introduction, we discuss different taxonomies of real-world scenes, and review a number of visual features that are thought to underpin human scene categorization. We discuss the behavioral and computational evidence that feature diagnosticity depends on taxonomy, and then outline the importance of establishing more rigorous taxonomies.

### Systems of categorization

Category systems can differ in their descriptive scope: a single environment might be described as “natural,” “ forest,” or “deciduous thicket.” Each description carries a different amount of detail. Tree hierarchies have been used to represent the multilevel organization of categories (Rosch, 1975; Rosch, 1999; Rosch & Lloyd, 1978; Rosch & Mervis, 1975; Tversky & Hemenway, 1983). Superordinate categories (e.g., natural vs. man-made or indoor vs. outdoor distinctions) are located at the highest tier of the hierarchy, basic-level categories describe variations within superordinate categories (e.g., mountain and coast are subdivisions of natural scenes) and subordinate categories capture finer distinctions within basic-level categories (e.g., pebbly beaches and sea cliffs within coastal scenes).

Rosch and Lloyd (1978) argue that “the task of category systems is to provide maximum information with the least cognitive effort” (p. 10) and propose that basic-level categories offer the most economical mode of description. Indeed, basic-level names are usually the default: we tend to describe a scene as a “forest,” avoiding coarser descriptions such as “natural,” or finer qualifications like “a coniferous forest in autumn” (Hajibayova, 2013). Basic-level categories purportedly offer an optimal trade-off between distinctiveness and informativeness (Murphy & Smith, 1982; Rosch & Lloyd, 1978; Tversky & Hemenway, 1983), and, unlike superordinate categories, may be encoded automatically or involuntarily in response to visual images (Greene & Li, 2014).

### Category systems and feature encoding

Although there seems to be a general preference for using basic-level categories, factors including stimulus presentation duration (Kadar & Ben-Shahar, 2012; Loschky & Larson, 2010), presentation order (Mack & Palmeri, 2015), and familiarity (Anaki & Bentin, 2009), can bias scene categorization toward superordinate or subordinate distinctions. Similarly, the “entry level” (i.e., most quickly accessed level) of object categorization is affected by stimulus typicality and the observer's subjective expertise (Johnson & Mervis, 1997; Jolicoeur, Gluck, & Kosslyn, 1984; Murphy & Brownell, 1985; Tanaka & Taylor, 1991).

**Figure 1.**
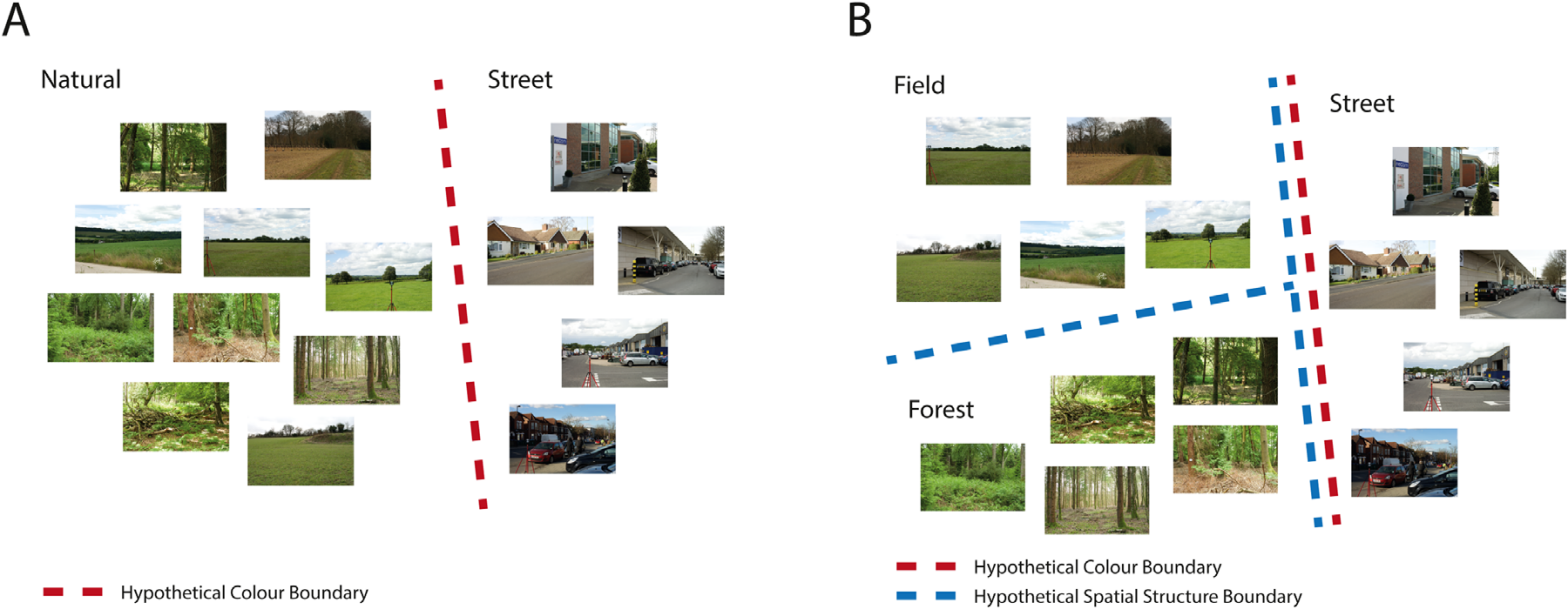
A toy example of how particular category structures can determine the visual features informative for discrimination. Color cues may be sufficient to discriminate natural images from street images (A), but less informative for discriminating subsets of natural scenes (B, field and forest categories). In this case, information about the spatial structure (among other features; see below for discussion) would be useful.

The ease of categorization may reflect where an image sits relative to the boundaries that carve out the “perceptual space” into distinct categories (Sofer, Crouzet, & Serre, 2015). Sampling images that maximize the distance to a relevant category boundary (e.g., natural vs. manmade), facilitates category discrimination (Sofer et al., 2015). Thus, the category system, in addition to individual differences, may alter the cues that are informative for categorization (Figure 1). In the related case of *object* categorization, encoding of background/context (Prass, Grimsen, Konig, & Fahle, 2013), orientation (Hamm & McMullen, 1998), and high spatial frequencies (Collin, 2006; Collin & McMullen, 2005) varies over different category systems (i.e., over different levels in the tree hierarchy of categories). As we explore in this article, similar effects have been observed for scene categorization. This interplay between perceptual coding and categorization highlights the importance of understanding the category systems that humans naturally use.

#### Objects

The hierarchical architecture of the human visual system suggests that complex perceptual representations are built from collections of simpler components or features. Early theories similarly held that scene recognition proceeds from an initial stage in which the identity and position of individual shapes or objects is determined (Bulthoff & Mallot, 1988; Hildreth, 1987; Marr, 1982; Watt, 1990). Experimental work suggests that object identification improves subordinate scene category discrimination (Collin & McMullen, 2005; Malcolm, Nuthmann, & Schyns, 2014). However, basic-level and superordinate scene categories are identified *in parallel* with object categories (Fabre-Thorpe, 2011; Joubert, Rousselet, Fize, & Fabre-Thorpe, 2007; Rousselet, Joubert, & Fabre-Thorpe, 2005; VanRullen & Thorpe, 2001), and computing superordinate or basic-level scene category from object statistics is computationally expensive (Greene, 2013). These results suggest that objects are more useful for subordinate categorization, possibly owing to stronger object predictability for subordinate categories, or redundancy between object identities and concurrently available low-level image features for coarser category discriminations (discussed elsewhere in this article). It has also been argued that objects may be more frequent and diverse in indoor scenes (Greene, 2013). As a result, empirical measurements of the utility of object identification for scene categorization may depend on the prevalence of indoor categories in the dataset or experiment.

#### Spatial layout

**Figure 2.**
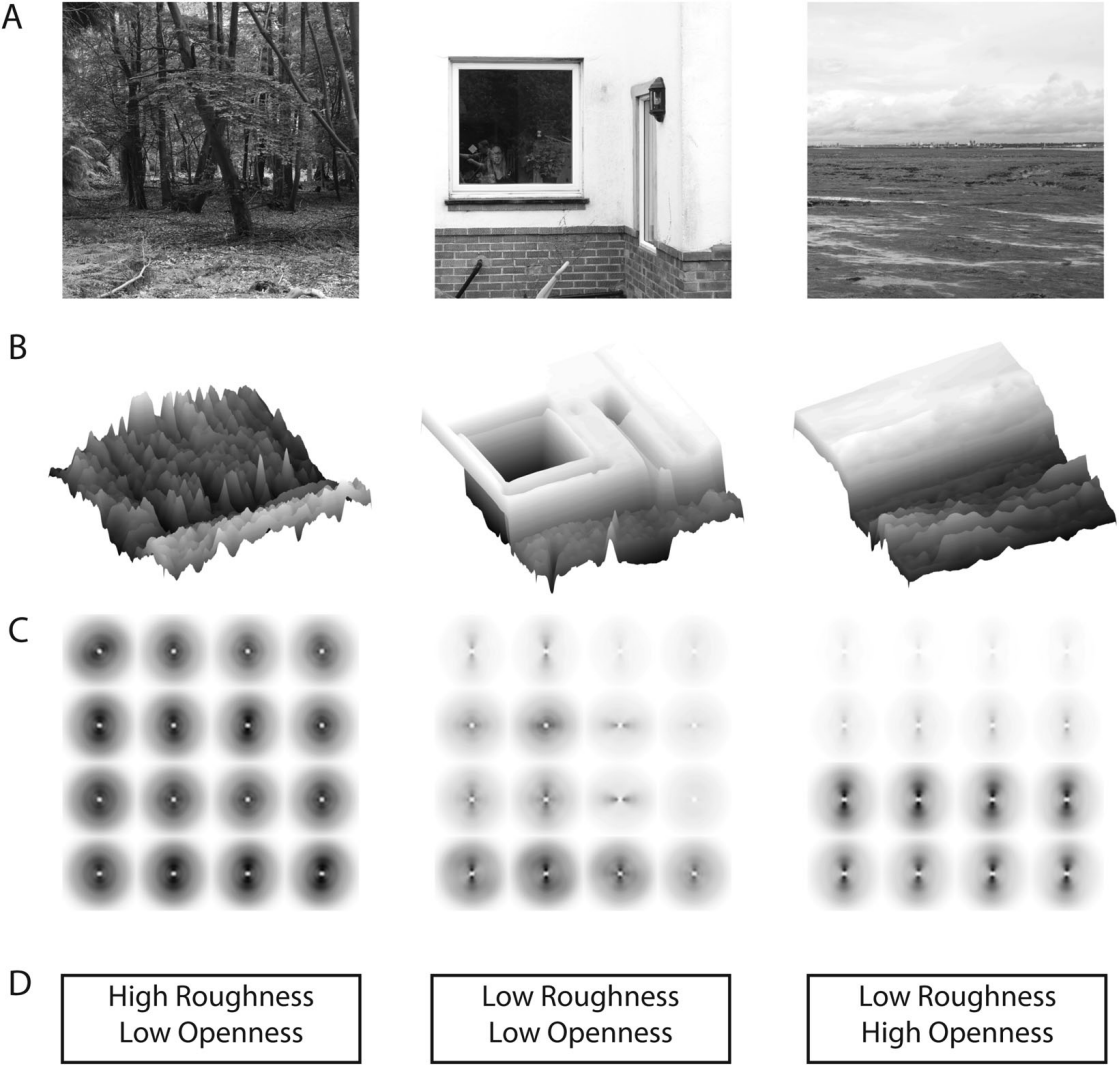
The spatial layout of natural scenes correlates with low-level global image features. (A) Images from the SYNS dataset (Adams et al., 2016). (B) Variations in pixel intensity across the image capture some characteristics of the scene's spatial structure without object segmentation. Height corresponds to pixel luminance (images were low-pass filtered with a gaussian kernel: bandwidth = 50 pixels). (C) Spectrograms provide a visualization of the distribution of low-level image features. High energy is indicated in each polar plot by dark regions. For example, in the image on the right, contrast energy is concentrated in the lower half of the image, where there are horizontal image structures of high spatial frequencies (note that the high-energy, dark regions in the lower Fourier plots are vertically oriented and close to the center of each plot). The GIST feature descriptor provides a summary of these image spectra. (D) Spatial envelope properties, such as roughness and openness, can be predicted from GIST. Figure adapted from Oliva and Torralba (2001).

According to the spatial envelope model (Oliva & Torralba, 2001, 2006; Torralba & Oliva, 2002, 2003), an image's semantic category (e.g., beach vs. forest) can be recovered using a small set of image descriptors termed spatial envelope properties (e.g., openness, naturalness, roughness) that represent the spatial layout of the scene (Figure 2). Classifiers trained to predict semantic categories from human-labelled spatial envelope properties perform similarly to humans (Greene & Oliva, 2006, 2009b). Moreover, adaptation studies suggest that human category representations rely on spatial envelope properties, or correlated features: after prolonged viewing of an image set with similar spatial envelope properties, subsequent categorization is biased away from the adaptation set (Greene & Oliva, 2010). For example, adaptation to images high in openness generates a bias toward low-openness categories such as forests. Spatial envelope properties may be computed from statistics of low-level visual features (e.g., histograms of edges or Fourier amplitude spectra) pooled over large areas of the visual field (Oliva & Torralba, 2001). For example, human-rated spatial envelope properties can be predicted by the GIST image descriptor proposed by Oliva and Torralba (2001). The GIST descriptor computes a histogram of average responses to Gabor-like filters at different orientations and scales over different spatial regions of an image (usually a 4 × 4 grid). Because global GIST features predict human-rated spatial envelope properties, which in turn predict semantic categories, a core tenet of the spatial envelope model is that category membership can be determined without parsing an image into its constituent objects (Oliva & Torralba, 2001).^1^

The spatial envelope model predicts that category systems that maximize between-category differences in coarsely localized GIST features will be discriminated more efficiently by humans. Although basic-level categories are thought to be encoded more or less automatically (as discussed elsewhere in this article), some work has shown that superordinate categorization in fact *precedes* basic-level categorization (Fabre-Thorpe, 2011; Kadar & Ben-Shahar, 2012; Loschky & Larson, 2010; Sun, Ren, Zheng, Sun, & Zheng, 2016). According to the spatial envelope model, this superordinate advantage emerges because superordinate categories are more separable in the GIST feature space than basic-level categories (Loschky & Larson, 2010; Oliva & Torralba, 2001). Indeed, if a biased sample of images maximizes the discriminability of basic-level members in GIST-space, basic-level categorization precedes superordinate categorization (Sofer et al., 2015). The spatial envelope model also predicts that GIST features discriminate man-made vs. natural categories better than indoor vs. outdoor categories (Oliva & Torralba, 2001), and behavioural work confirms that natural vs. man-made category distinctions are faster than indoor vs. outdoor distinctions (Banno & Saiki, 2015; Kadar & Ben-Shahar, 2012). In other words, not all superordinate categories are distinguished equally easily, and this could reflect differences in the perceptual availability of discriminative spatial layout information.

#### Affordances

An alternative, affordance-centered account of category representations emphasizes that the “conceptual structure of environments is driven primarily by the scene's *functions*, or the actions that one could perform in the scene” (Greene, Baldassano, Esteva, Beck, & Li, 2016; Groen et al., 2018). Greene et al. (2016) suggest that scene categories are better predicted by functional information than features such as color, spatial layout, attributes (surfaces, materials, etc.), object co-occurrence statistics, and so on. Importantly, however, affordances necessitate objects to be acted upon, or spaces to be acted within—and thus rely on extracting objects and spatial structure. Moreover, though Greene et al.'s (2016) affordances are stronger predictors of human categorization than categorization models based on GIST, or other low-level image statistics (e.g., Oliva & Schyns, 2000; Oliva & Torralba, 2001), the latter models were expressly formulated to reveal the diagnostic features of early visual representations and were tested on superordinate or basic-level categories (e.g., the spatial envelope model was formulated to discriminate eight basic level categories; Oliva & Torralba, 2001). In contrast, Greene et al. (2016) tested these models on 311 subordinate categories. Low-level image statistics may be more useful for basic- or superordinate-level scene categorization, whereas subordinate-level scene categorization may be more closely related to affordances.

#### Color

The greenness of forests, blueness of coastlines, and yellowness of deserts are highly predictive low-level features for categorization (Goffaux et al., 2005). Abnormally colored scenes (e.g., a beach scene with a yellow sky and blue sand) that contain the same color segmentation cues as normal scenes (i.e., with similar discontinuities in hue at object/surface boundaries) take longer to categorize (Castelhano & Henderson, 2008; Goffaux et al., 2005; Oliva & Schyns, 2000). Hence, color improves categorization not only because it may benefit segmentation, but because some categories have well-defined color profiles. Color-based improvements are larger for indoor vs. outdoor urban discriminations than natural vs. manmade discriminations (Rousselet et al., 2005), presumably because artificially illuminated indoor scenes tend to be more “yellowish/brownish” than either natural or manmade outdoor scenes (Rousselet et al., 2005). Computational work confirms that color cues reliably discriminate indoor vs. outdoor images (Szummer & Picard, 1998; Tong, Shi, Yan, & Wei, 2017). Clearly, the benefit of color information varies with the distribution of colors within and between category representations.

In this brief review of four feature dimensions (objects, spatial structure, affordances, and color), we have highlighted how the cues informative for categorization depend on the taxonomical structure of the chosen category system. This dependence highlights the importance of understanding the actual scene taxonomies that humans rely on when viewing real-world scenes. Next, we discuss the strengths and weaknesses of existing approaches to taxonomizing real-world scenes.

### Existing scene taxonomies

Large-scale databases such as ImageNet (Deng et al., 2009), Places (Zhou, Lapedriza, Xiao, Torralba, & Oliva, 2014), and SUN (Xiao et al., 2010) have used WordNet (Miller, 1995) to identify quasi-exhaustive dictionaries of category terms. These terms are then entered into search engines to collect image stimuli. However, the fine granularity of WordNet labels is atypical of human language: humans show up to 32.7% disagreement regarding the meaning of these labels (Chklovski & Mihalcea, 2003). WordNet was built by expert lexicographers and many terms require substantial esoteric knowledge. For example, “dolmen,” “medina,” “indoor cloister,” “mastaba,” and “oast house” are all categories from WordNet used in the SUN and Places databases (Xiao et al., 2010; Zhou et al., 2014). Computational work has shown that merging these fine-grained representations into larger clusters improves word-sense disambiguation (i.e., the identification of the correct meaning of a word, given multiple meanings—e.g., “bass”; Navigli, 2006; Snow, Prakash, Jurafsky, & Ng, 2007), and recent behavioral work suggests that humans integrate these senses into simpler taxonomies with fewer categories (Greene, 2019).

Additional problems may stem from the putative interchangeability of category terms such as “coast,” “beach,” and “seaside.” Well–documented effects of cognitive-linguistic categories on early visual processing suggest that different category labels may elicit different visual representations, and different categorization behavior (e.g., Bentin & Golland, 2002; Goffaux, Jemel, Jacques, Rossion, & Schyns, 2003; Schyns & Oliva, 1999). Semantic labels modulate low-level visual representations within 44 to 150 ms of stimulus onset (Boutonnet & Lupyan, 2015; Maier, Glage, Hohlfeld, & Rahman, 2014; Noorman, Neville, & Simanova, 2018)—a time window in which important scene properties such as color and spatial structure are encoded (Cichy, Khosla, Pantazis, & Oliva, 2017; Goffaux et al., 2005; Ramkumar, Hansen, Pannasch, & Loschky, 2016). Thus, it is important to establish the category labels that participants would most frequently or naturally use.

### Main research question

We have argued that the taxonomical structure of category systems used in empirical perceptual research can undesirably confound scene perception and categorization responses. To address this problem, we aim to develop a method to identify the categories that humans most naturally use to taxonomize visual environments. In Experiment 1, we present a novel method to derive ground-truth category systems from human grouping judgements in a flexible image sorting and labelling task that minimizes instruction and experimenter bias. We present category systems for three dimensions: semantics, three-dimensional (3D) spatial structure, and two-dimensional (2D) appearance. In Experiment 2, we label a larger number of images from the Southampton-York Natural Scenes (SYNS) dataset using these categories, and examine the generalizability of the categories derived from Experiment 1. We also explore the relationships between generated categories across the three dimensions. In Experiment 3, we examine the relationship between our category systems and the spatial envelope model. Finally, in Experiment 4, we evaluate our method on a larger and completely independent dataset, using a different experimental paradigm.

## Experiment 1

### Methods

#### Participants

A convenience sample of 24 naïve undergraduate and postgraduate students, 19 female, age range: 18–26 years, from the University of Southampton participated as volunteers, or in return for course credits. Each of the three tasks (semantic, 3D spatial structure, 2D appearance) was completed by 20 participants (individual participants completed two or three tasks each; the order was counterbalanced). For all experiments, informed consent was obtained before experimentation, and ethical approval was acquired from the Research Governance Office, University of Southampton.

#### Materials

Eighty full-color stereo-pairs (one randomly sampled pair from every scene) were sampled from the SYNS database (Adams et al., 2016). Stimuli were presented on a dual-monitor display (two 32-inch, 2560 × 1440, 75-Hz, ASUS PB328Q monitors) via a single-bounce Wheatstone mirror stereoscope at an effective viewing distance of 83.5 cm. Stimuli were presented en masse as monoscopic thumbnails (3.98 × 2.64° of visual angle), but observers selected individual images for enlarged stereoscopic viewing. (Grouping was performed using stereoscopic images to capture the role of binocular depth cues in scene perception.) The stereoscopic images were displayed at 31.12 × 22.36° of visual angle. Every participant viewed the same images. The entire task was programmed in MATLAB (MathWorks, Inc., Natick, MA).

#### Procedure

Participants sorted images into discrete categories. This task was completed separately for each of the three dimensions. Task instructions informed participants of the grouping system they would use (see Appendix for full instructions).

##### Semantic task

Images were grouped by the “type of place” (e.g., mountain).

##### Three-dimensional spatial structure task

Images were sorted according to their depth structure. Participants were encouraged “to think about the 3D model that you would have to physically build to represent each scene” and to consider how the physical structure of some scenes might be similar or different.

##### Two-dimensional appearance task

Images were grouped by their 2D appearance (ignoring variations in 3D structure). Participants were instructed to attend to the “colors, patterns . . . or textures,” materials, luminosity, etc., (e.g., blue or red).

In every task, participants were urged to consider each image in its entirety, and discouraged from focusing on smaller subregions like individual objects. Participants were limited to between three and 10 categories. This constraint served as a liberal middle-ground between accepted set sizes of superordinate (two to three; e.g., Fei-Fei et al., 2007; Oliva & Torralba, 2001), and basic-level categories (seven to 13; e.g., Fei-Fei & Perona, 2005; Vailaya, Jain, & Zhang, 1998). Although there are undoubtably a larger number of possible categories than our limit of 10, the SYNS dataset only contains a subset of all possible outdoor scenes (Adams et al., 2016). Categories could contain a minimum of two images.

**Figure 3.**
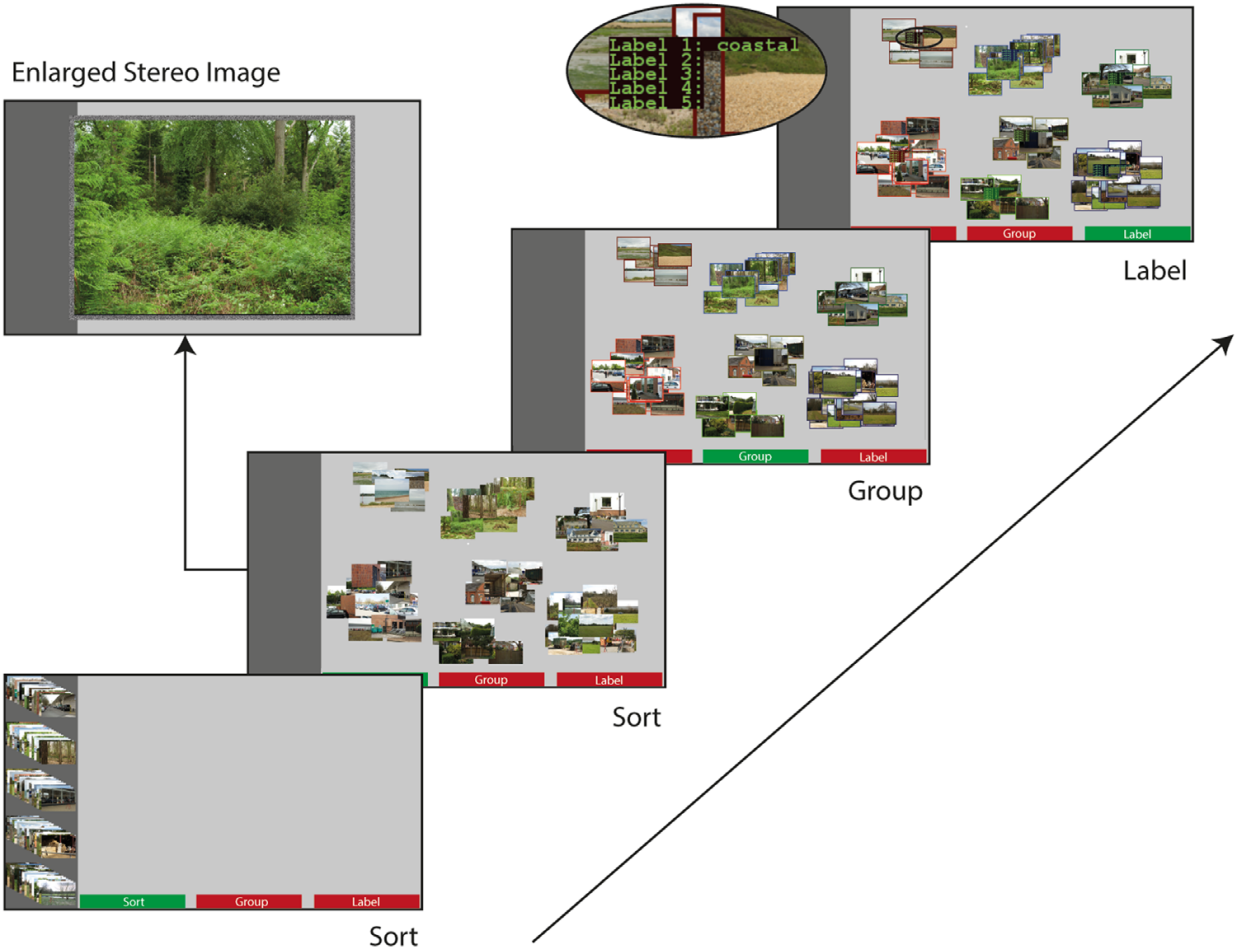
Schematic of the categorization task. Images were initially presented in five stacks of images (randomly assigned) on the left margin of the display. Sort: Participants dragged and dropped each image into the workspace, stacking same category images. Enlarged, stereoscopic versions of each image could be viewed at any time. Group: Participants checked the validity of all categories and returned to the Sorting stage if any categories contained only one image or if the number of categories fell outside the range of three to 10. Label: Participants labelled each category.

Each task contained three activities: “Sort,” “Group,” and “Label” (Figure 3). Participants accessed each activity by clicking corresponding tabs at the bottom of the display using the mouse.

##### Sort

All 80 images were initially stacked in random order on the left margin of the display. Participants created categories by dragging images, one at a time, into the workspace; categories were defined as any set of overlapping images. Enlarged stereoscopic versions of the images were viewed by simultaneously pressing two mouse buttons.

##### Group

Category validity was automatically checked. If participants generated fewer than three or more than 10 groups, a thick black frame highlighted all categories. If any group contained fewer than two images, the invalid category or categories were highlighted. Valid groups were each highlighted with a differently colored border. Participants continued to the “Label” stage once all categories were valid.

##### Label

Participants typed between one and five labels to describe each group of images.

Participants had unlimited time to complete each categorization task and took an average of 30 minutes per task.

#### The CIRCA method

We developed the clustering by increasing the rand index via coordinate ascent (CIRCA) method to organize images into categories based on psychophysical judgements of stimulus similarity. In an experiment that generates pairwise similarity responses, such as the sorting experiment described elsewhere in this article, it is possible to represent each participant's data using an *n* x *n* similarity matrix that codes the pairwise similarity between all images by a series of 1s and 0s (1 if a given pair were placed into the same group, and 0 if they were not). Averaging these matrices across participants gives ***S***, a similarity matrix that codes the average association between every image pair. Our ultimate aim is to identify the set of categories that maximizes in-group similarities and minimizes out-group similarities in ***S***.

The Rand index quantifies the agreement between two sets of categories by summing i) the number of pairs that are in the same category in both sets, and ii) the number of pairs that are in different categories for both sets, and dividing by the total number of pairs (Rand, 1971). A score of 1 represents perfect agreement between two sets of categories, and 0 represents no agreement.

Because the Rand index quantifies the agreement between sets of hard categories (where each datapoint belongs to only one category), and ***S*** represents category membership on a continuum, we adapted the Rand index to determine α, the agreement between the similarity matrix ***S*** and a proposed clustering ***c*** = *c*_1_, *c*_2_,…*c_n_*, where *c_i_* represents the category to which image *i* has been assigned. Let *s*_*ij* _be the (*i*, *j*)th element of ***S***, measuring the similarity of images *i* and *j*. Then, we define the affinity between ***c*** and ***S*** as:

(1) $$\begin{array}{c} \;\;\;\;\;\;\;\;\;\;\;\;\;\alpha =\frac{1}{n(n-1)/2}\sum_{j>i}\left(c_{i}=c_{j}\right)\;s_{ij}+\left(c_{i}\neq c_{j}\right)\left(1-s_{ij}\right)\; \end{array}$$

Our goal is to find the clustering *c* that maximizes the affinity α. We maximize α by iterative coordinate ascent from a random initial clustering. On each iteration, reassignment of a randomly selected image to a randomly selected category is proposed. Proposals that increase α, and therefore improve the agreement between *S* and *c*, are accepted. This process is repeated until no move increases α (i.e., until a stationary point is reached). Because it is possible for our method to converge at local maxima, this entire procedure is repeated from a number of different starting positions (initial clusterings).

Given *n* stimuli and *k* clusters, there are *k^n^* possible clustering solutions, and, on every iteration of coordinate ascent, the maximum number of proposals before a single reassignment is *n*(*k* − 1). We empirically tested the time complexity of our method on simulated datasets of various sizes, and found that time-to-convergence (i.e., stationarity) increases linearly as a function of *n*, and increases with *k* following a power law (see Supplementary Materials, Supplementary Figure S1).

To find the globally optimal clustering, our method can be implemented multiple times for different numbers of clusters. To protect against overfitting, we cross-validate clusterings on left-out data using the adjusted form of the Rand index (ARI), which controls for variation in chance-level agreement as a function of the number of clusters (Hubert & Arabie, 1985).^2^ If the validation data are a hard clustering, then the ARI is calculated as in Hubert and Arabie (1985), but if the validation data is a soft clustering (e.g., an average of responses from multiple observers), which has an undefined number of clusters, then the adjustment to the Rand index can be calculated by simulating the agreement between the validation set, and a random clustering with the same number of clusters as the model. The soft clustering formulation of the ARI is then:

(2) $$ARI=\frac{RI_{m}-RI_{r}}{1-RI_{r}}$$

where *RI_m_* is the rand index from the model, and *RI_r_* is the rand index from the random clustering.

A comparison against popular alternative clustering algorithms (k-medoids and spectral clustering), reveals that our method is more robust against response noise (Supplementary Materials, Supplementary Figure S2). Moreover, we show that our method tolerates high levels of interobserver disagreement, and reproduces the exact clustering given an internally consistent set of similarity judgements (Supplementary Figure S3). The code for the MATLAB implementation of this algorithm is available at: *https://github.com/mattanderson94/CIRCA_Clustering*.

#### Statistical analyses

For the semantic, 3D spatial structure, and 2D appearance sorting tasks, we identified the category system that best represented the grouping judgements of all participants. To this end, we i) identified the optimal number of categories, ii) determined the optimal category for each image, and iii) selected names for each category from participants’ labels. We describe our method of solving each of these problems in turn.

##### Identifying the optimal number of categories

First, for a given task, we identified the optimal number of categories using the CIRCA method. We considered clusterings with between *k* = 1:20 distinct categories. To avoid overfitting, we used leave-one-out cross validation (LOOCV) over our 20 participants, leaving each participant out in turn and calculating the averaged 80 × 80 similarity matrix from the remaining 19 participants. We applied our method 1,000 times (i.e., from 1,000 different random initial clusterings) to find the clustering that produced the highest agreement with the left-out participant (measured using the ARI). The optimal number of categories was then identified as the *k* that produced the maximum ARI between the optimized clustering and left-out participants’ data, averaged across all (left-out) participants.

##### Defining the optimal group-level solution

Having identified the optimal *number* of categories, we determined the optimal group-level clustering using the CIRCA method on a similarity matrix based on the data from *all* 20 participants. (Here, we used 10,000 random initializations.)

##### Assigning participant-generated labels to each category

Next, we assigned labels to the optimal group-level categories. We used the ARI to quantify the agreement between every group-level category and every raw participant-generated category (and associated labels) while holding all other participant-generated and group-level categories constant. Consider, for example, a participant that constructed four categories. First, we isolate category 1—and partial out the rest—2, 3 and 4—by assigning them all to a common, second category, and then we apply the same treatment to the group-level categories. The ARI determines how well the selected participant's category (and associated label) describes the selected group-level category. ARIs for categories with matching labels from different participants (i.e., multiple uses) were summed. Pluralisms, nouns, adjectives, and verbs with a common stem were treated as the same—for example, one observer might have used the label “Farms,” and another observer, “Farm,” or “Farming.” The “winning” label with the greatest summed ARI was assigned to each category.

To ensure that the final labels represented all images in the category, a secondary label was assigned to a category where it i) conferred novel meaning beyond the primary label, *and* ii) was strongly associated with the images within the category. To quantify requirement (i), we determined the semantic similarity between the primary label (i.e., the label with the greatest ARI per category), and every other label using spaCy v2.0 (https://demos.explosion.ai/similarity/). Labels with similarity scores of less than 0.50 were deemed sufficiently low to capture a new or different meaning. For example, “Beach” and “Seaside” describe semantically overlapping concepts (semantic similarity = 0.71) and thus provide redundant information, whereas “Car Park” and “Commercial” (semantic similarity = 0.43) refer to different scene types. Requirement (ii) was met by normalizing per-label ARIs to range from 0 to 1, and rejecting values of less than 0.65.

Data can be downloaded from: https://doi.org/10.5258/SOTON/D1649.

### Results

**Figure 4.**
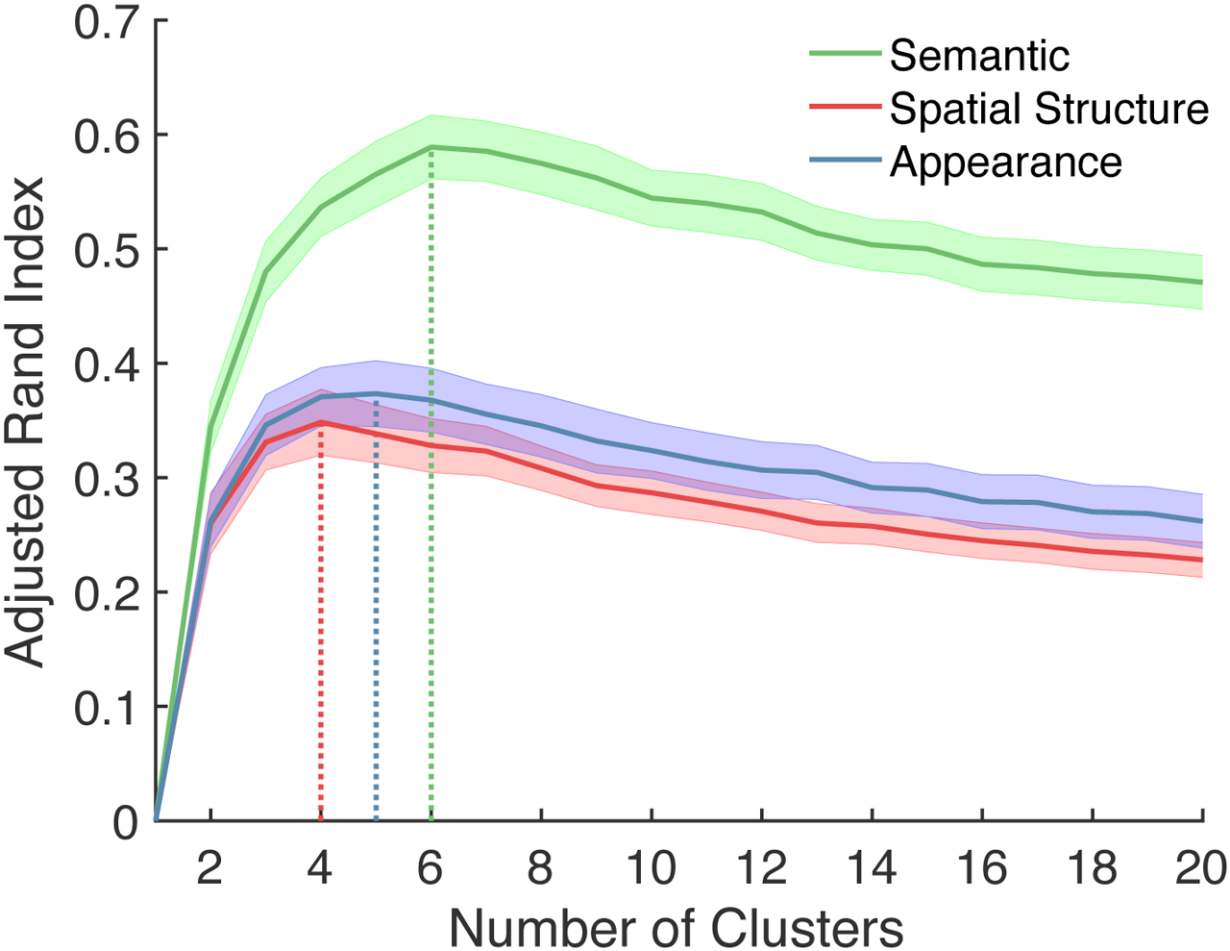
The optimal number of categories per dimension was determined via LOOCV. The *y*-axis gives the maximum ARI averaged over 20 participants (from 1,000 random initializations of LOOCV) as a function of the number of clusters, *k* (*x*-axis). Shaded regions represent ±1 participant standard error. Vertical dashed lines identify the global maximum for each dimension.

Figure 4 summarizes the results of the LOOCV analyses used to identify the optimal number of categories. Within each task or dimension, a single peak in the average ARI can be observed: the optimal number of categories for the semantic, 3D spatial structure, and 2D appearance categories were six, four, and five, respectively (vertical dashed lines). Interobserver agreement (as indexed by agreement between the derived group-level categories and each observer's data) was substantially higher in the semantic task (*ARI* = 0.59) than the 3D spatial structure (*ARI =* 0.35) and 2D appearance tasks (*ARI* = 0.37).

#### Semantic categorization

**Figure 5.**
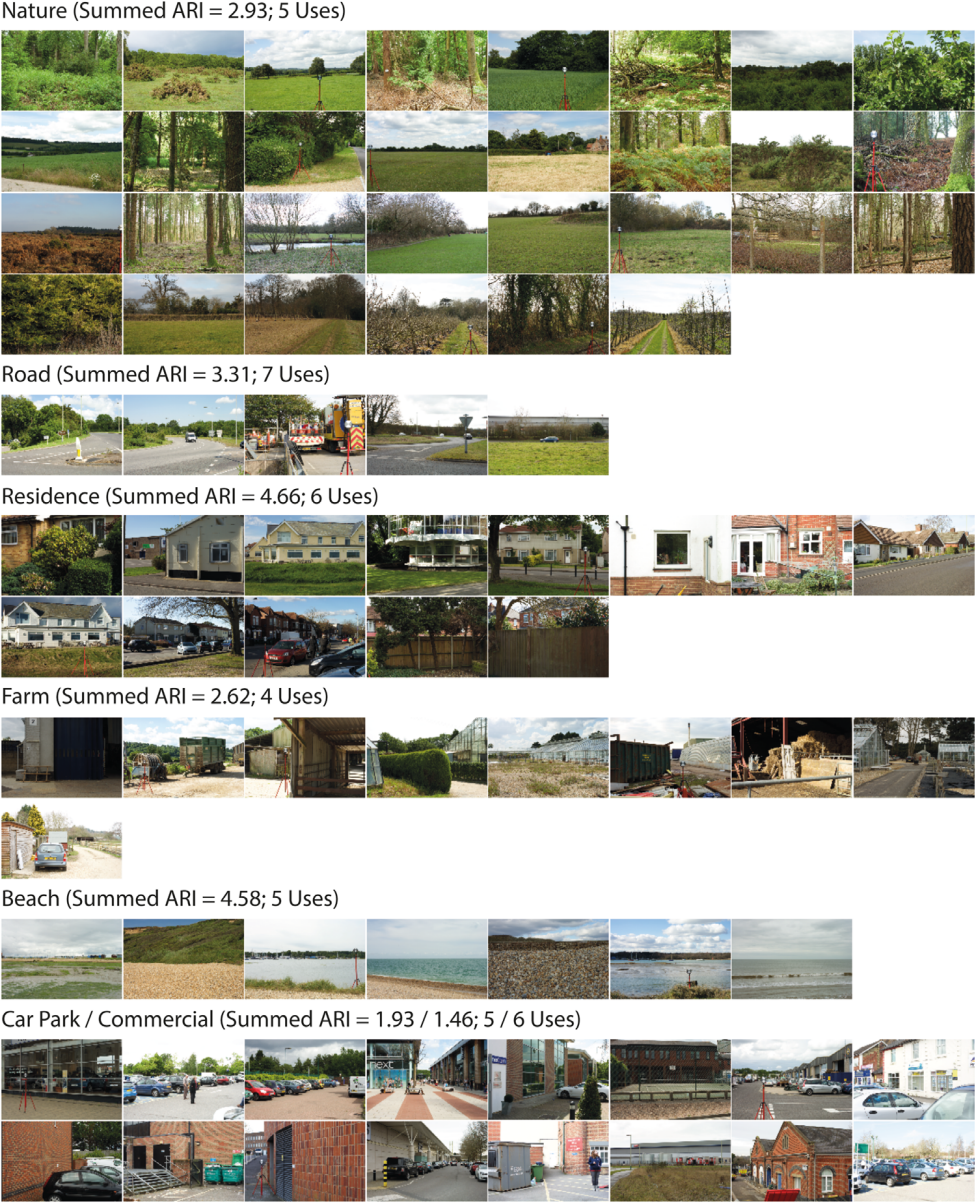
Images assigned to the six optimal semantic categories. Above each category we present the category labels, which were derived by summing the ARIs over the multiple uses across different participants, and picking the maximum/maxima.

The images associated with each group-level derived category are presented in Figure 5, with the optimal label(s). The category labels are “Nature,” “Road,” “Residence,” “Farm,” “Beach,” “Car Park,” and “Commercial.”

#### Three-dimensional 3D spatial structure categorization

**Figure 6.**
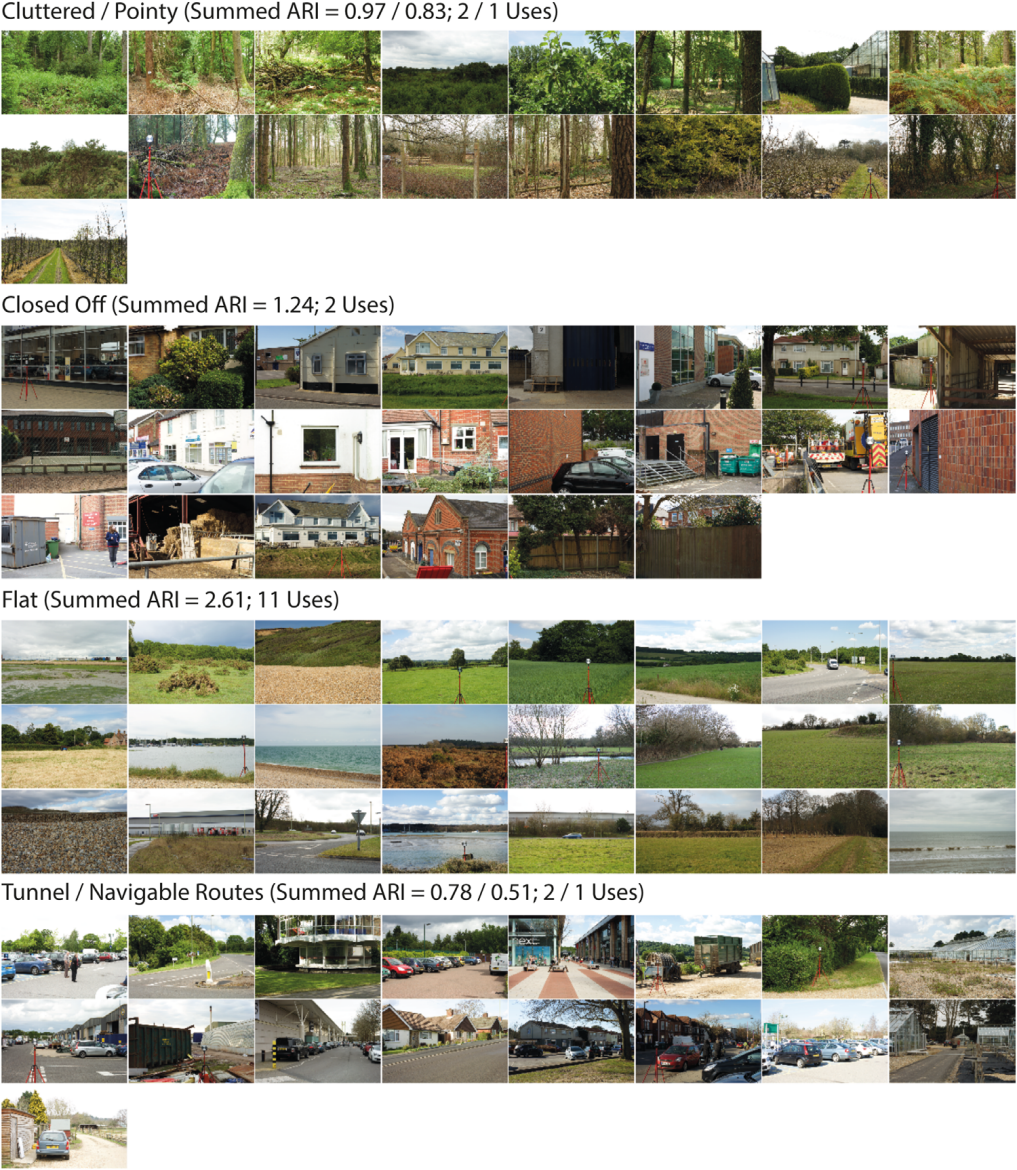
Images assigned to the four optimal 3D spatial structure categories. Above each category, we present the category labels, which were derived by summing the ARIs over the multiple uses across different participants, and picking the maximum/maxima.

The optimal 3D spatial structure categories are presented in Figure 6. The category labels are “Cluttered” or “Pointy,” “Closed Off,” “Flat,” and “Tunnel” or “Navigable Routes.”

#### Two-dimensional appearance categorization

**Figure 7.**
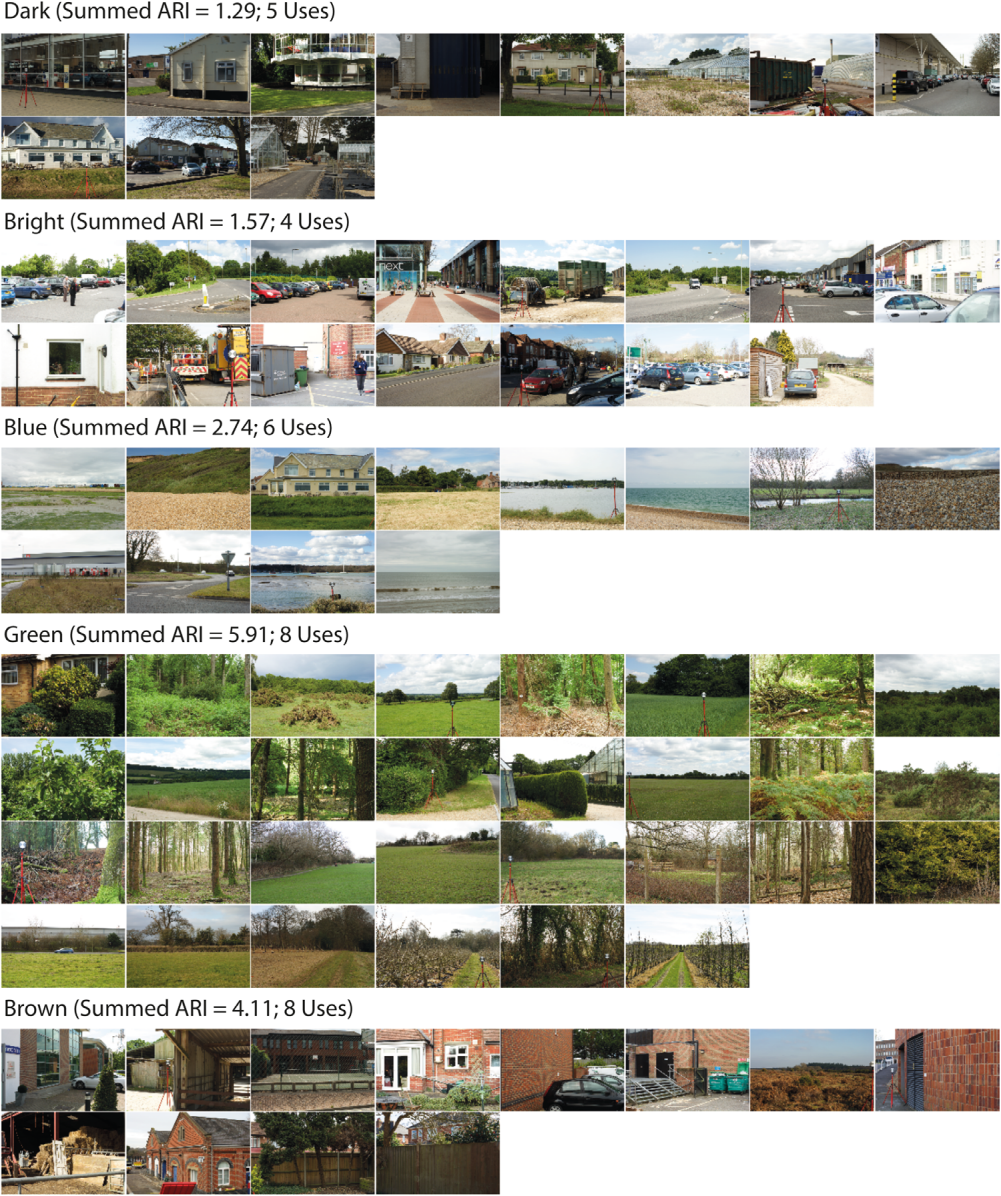
Images assigned to the five optimal 2D appearance categories. Above each category we present the category labels, which were derived by summing the ARIs over the multiple uses across different participants, and picking the maximum/maxima.

The optimal 2D appearance categories are presented in Figure 7. The category labels are: “Dark,” “Bright,” “Blue,” “Green,” and “Brown.”

### Discussion

Experiment 1 included only 80 images—a manageable number for our grouping task. In Experiment 2, we asked observers to use the labels derived in Experiment 1 to categorize a larger set of images from the SYNS dataset. We then i) test how well the categories developed in Experiment 1 generalize to the new stimuli and new observers, and ii) evaluate the relationships between the category members across our category systems for the three dimensions.

## Experiment 2

### Methods

#### Participants

Thirty-three naïve undergraduate and postgraduate students, 27 female; age range: 18 to 23 years, from the University of Southampton, none of whom participated in Experiment 1, were recruited as volunteers, or in return for course credits. Twenty completed the semantic categorization task, 20 completed the 3D spatial structure task, and 20 completed the 2D appearance task (participants performed one or two tasks each; the order was counterbalanced). Informed consent was obtained before experimentation, and ethical approval was acquired from the Research Governance Office, University of Southampton.

#### Materials

For each of the 80 outdoor scenes in the SYNS database (Adams et al., 2016), 18 stereo pairs compose a 360° panorama of each environment. Adjacent stereo pairs overlap, so we selected every other image—nine from each scene—to obtain a total of 720 images. Participants viewed full-size stereoscopic images, subtending 31.12 × 22.36° of visual angle (the same size as the large-scale images in Experiment 1).

#### Procedure

Separately for semantics, 3D spatial structure, and 2D appearance, participants classified every image according to the category labels derived in Experiment 1. Participants viewed one image at a time and used a mouse to select the appropriate category label from the list displayed to the side of the image. Once a label was chosen, participants continued to the next image or trial. Participants categorized all 720 images. The image order was randomized between participants.

### Results

Per-image category membership was determined by the most frequently selected category label. To quantify how well the categories derived in Experiment 1 generalized to a separate group of participants, and a separate set of images, we examined interparticipant agreement for the 80 images used in Experiment 1, and the 640 remaining images (Table 1). Category judgements for the 80 images from Experiment 1 showed high agreement across the new participants in all three category systems. As in Experiment 1, agreement was greatest in the semantic task. This result thus shows that our category systems generalize well to new observers.

**Table 1.** Average interparticipant agreement in Experiment 2 by category systems (columns) and image subset (rows).

	Semantic	3D spatial structure	2D appearance
Chance	16.67%	25%	20%
80 images from Experiment 1	82.19%	70.19%	71.13%
Remaining 640 images	82.30%	71.71%	68.19%

Agreement for the new set of 640 images was similar to that for the original 80 images from Experiment 1. Note, however, that for each new image, there is an image from Experiment 1 taken from the same location, but with a nonoverlapping field of view. This result thus shows that our category systems generalize well to new images, but it remains uncertain how well they will generalize to entirely new locations.

#### Intercategory relationships

**Figure 8.**
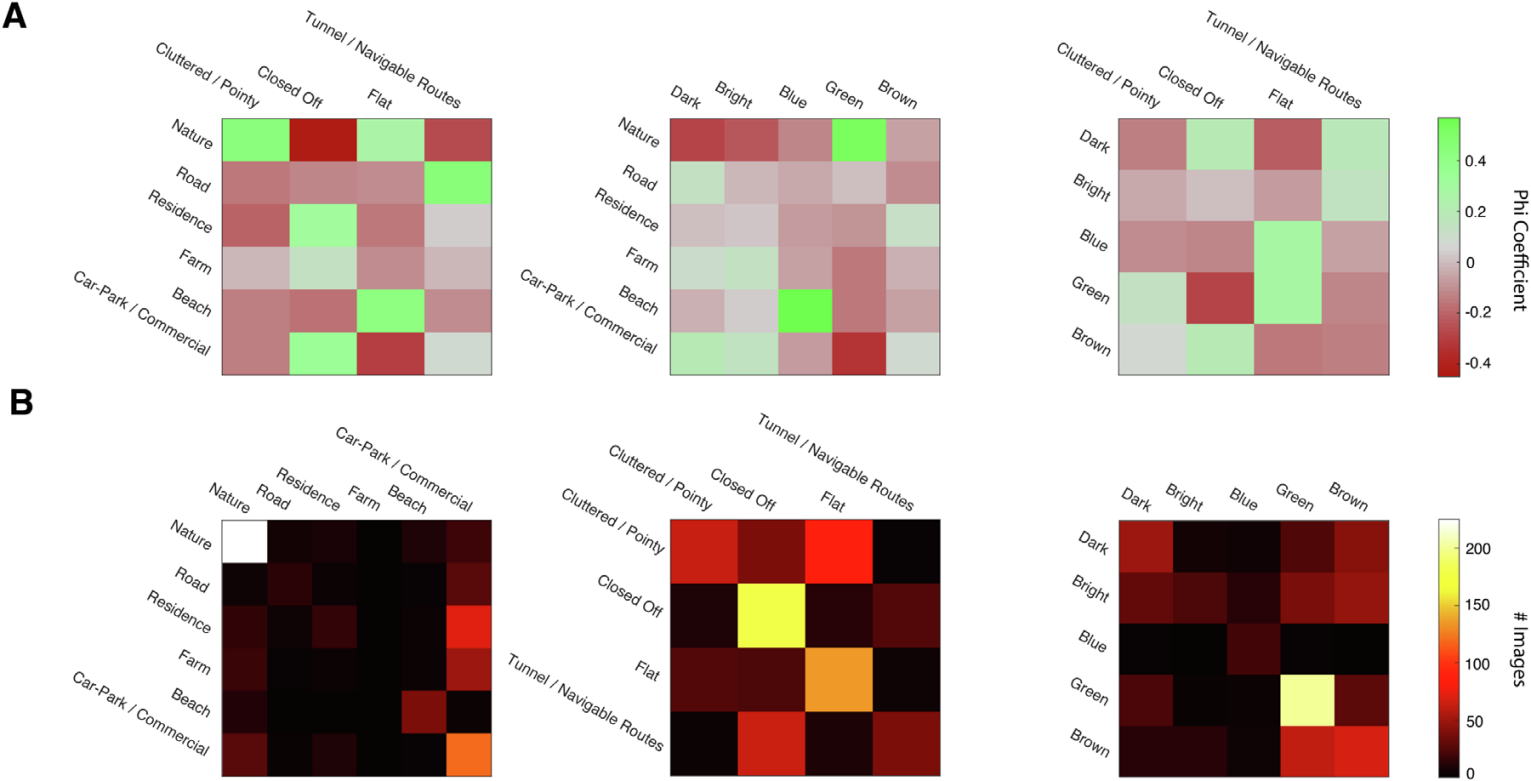
We examined the strength of the relationship between categories from different dimensions by (A) computing the phi coefficients between different categories and (B) testing the performance of a Bayes classifier trained to predict the category of an image, via leave-one-out cross-validation. In A, the left, middle, and right panels show the association between semantics and spatial structure, semantics and 2D appearance, and spatial structure and 3D appearance respectively. In B, confusion matrices show the predictions from three non-naïve Bayes classifiers: Spatial structure and 2D Appearance →  Semantics (left), Semantics and 2D Appearance →   Spatial Structure (middle), and Spatial structure and Semantic →   2D appearance (right). Rows are model predictions and columns are the true categories.

Phi coefficients (*r_φ_*) quantify the Pearson correlation between images with binary-coded categorical identity (images were either a member or not a member of a specified category). Positive values correspond to high categorical similarity (images were frequently placed in both categories), and negative values correspond to low categorical similarity (images frequently placed in one category were seldom placed in the other category). Figure 8A illustrates the intercategory correlations for our three category dimensions. Using this metric, intuitive intercategory relationships emerge (e.g., Nature and Green, Beach and Blue, Residence and Closed Off, and so on, are all positively correlated).

Next, we determined whether the relationships between category systems for the three dimensions were sufficient to drive reliable classification. In other words, we asked whether we can predict an image's category in one dimension from its category in one or both of the other dimensions. To explore this hypothesis, we used Bayes classifiers trained and tested via LOOCV. Table 2 presents the average classification accuracy over 720 left-out images for every combination of the category systems.

**Table 2.** Bayes classification accuracy per dimension. Classifiers tested using LOOCV on individual images.

	Predictor dimension(s)						
Predicted dimension	Chance	Prior-only	Semantic	Structure	Appearance	Both (naïve)	Both (non-naïve)
Semantic	16.67%	37.36%	–	53.47%	49.72%	55.97%	57.36%
Structure	25.00%	31.53%	60.00%	–	44.72%	58.19%	57.92%
Appearance	20.00%	35.69%	43.75%	42.22%	–	45.83%	50.56%

Reliable relationships between category membership across the three dimensions are indicated by the fact that all classifiers performed better than chance (1/*k*), and better than a prior-only model in which the most prevalent or probable category is always selected. The two-predictor classifiers outperformed the single-predictor classifiers, with the exception of 3D spatial structure, which was more accurately classified from semantic structure alone than both semantics and 2D appearance.

Accuracy alone offers a limited picture of the behavior of these models. Consequently, for each of the non-naïve classifiers, we plot confusion matrices between the true categories and predicted categories (from two predictors). The results are shown in Figure 8B. We found that categories vary substantially in difficulty. The semantic classifier accurately discriminated most “Nature” images, and produced reasonable predictions for “Car Park” images, but performed much more poorly on the other categories. A similar picture emerges for spatial structure classifier, which discriminated only the “Closed Off” and “Flat” categories well, and for the 2D appearance classifier, which discriminated only the “Green” categories well. These results indicate that the relationships observed between the three category systems may be limited to a *subset* of categories; not all categories are equally predictable.

We then explored two different ways to combine the two predictors. Let $C_{j}$ represent the set of possible categories for dimension *j* and *C_ij_* represent the category of image *i* in this dimension. In a naïve Bayes model, we assume that the two predictors are independent and factor the likelihoods. For example, when predicting the category membership for dimension one from dimensions two and three, we compute:

(3) $$C_{i1}=\underset{c\in \;CC_{1}}{\arg\;\max}p\left(C_{i1}=c|C_{i2}\right)p\left(C_{i1}=c|C_{i3}\right)$$

In our non-naïve Bayes model, we do not assume independence, and use the joint distribution:

(4) $$C_{i1}=\underset{c\in \;CC_{1}}{\arg\;\max}p\left(C_{i1}=c|C_{i2},C_{i3}\right)$$

The non-naïve Bayes model performed better than the naïve Bayes model when predicting category membership within two of the three dimensions, and only marginally worse for the third (3D spatial structure). This reveals a non-trivial interdependence between the classification systems for each dimension.

To assess the consistency of this interdependence across participants we tested naïve and non-naïve classifiers using LOOCV on *N* – 1 participants*,* evaluating how well each classifier predicted the left-out human categorization judgements (see Table 3). Again, predictions were well above chance and prior-only predictions, and the non-naïve Bayes model performed better than the naïve Bayes model for two of the three category systems, and only marginally worse for the third (semantic). This shows that the interdependence between classification systems is relatively stable across participants.

**Table 3.** Naïve and non-naïve LOOCV (on individual participants) Bayes classification accuracy per dimension (i.e., category system).

Predicted dimension	Prior-only	Semantic	Structure	Appearance	Naïve	Non-naïve
Semantic	37.36%	–	42.27%	44.65%	48.44%	48.08%
Structure	31.53%	15.33%	–	30.58%	26.05%	50.69%
Appearance	35.69%	39.49%	33.24%	–	39.60%	41.27%

#### Typical exemplar classification

Typical category instances can be defined as images with high interparticipant agreement; atypical images can be defined by low interparticipant agreement, that is, they are associated with multiple categories. Typical exemplars have a special status in category representations: they share many features with other members of the same category, and few with members of other categories (Rosch & Mervis, 1975). Global image features—including color and spatial structure—are more predictive of typical category exemplars than atypical category members (Ehinger, Xiao, Torralba, & Oliva, 2011; Torralbo et al., 2013).

We examined classification accuracy as a function of typicality by selecting the 30 images from each category with the highest interparticipant agreement. This produces a uniform prior, such that chance and “prior-only” performance is equated across categories as 1/*k*. Classification accuracy for these typical images was compared against accuracy for two other images subsets: one consisting of 30 *atypical* images, that is, those with the lowest interparticipant agreement per category, and one consisting of 30 randomly selected images per category. Once again, we used LOOCV to train and test each classifier.

**Figure 9.**
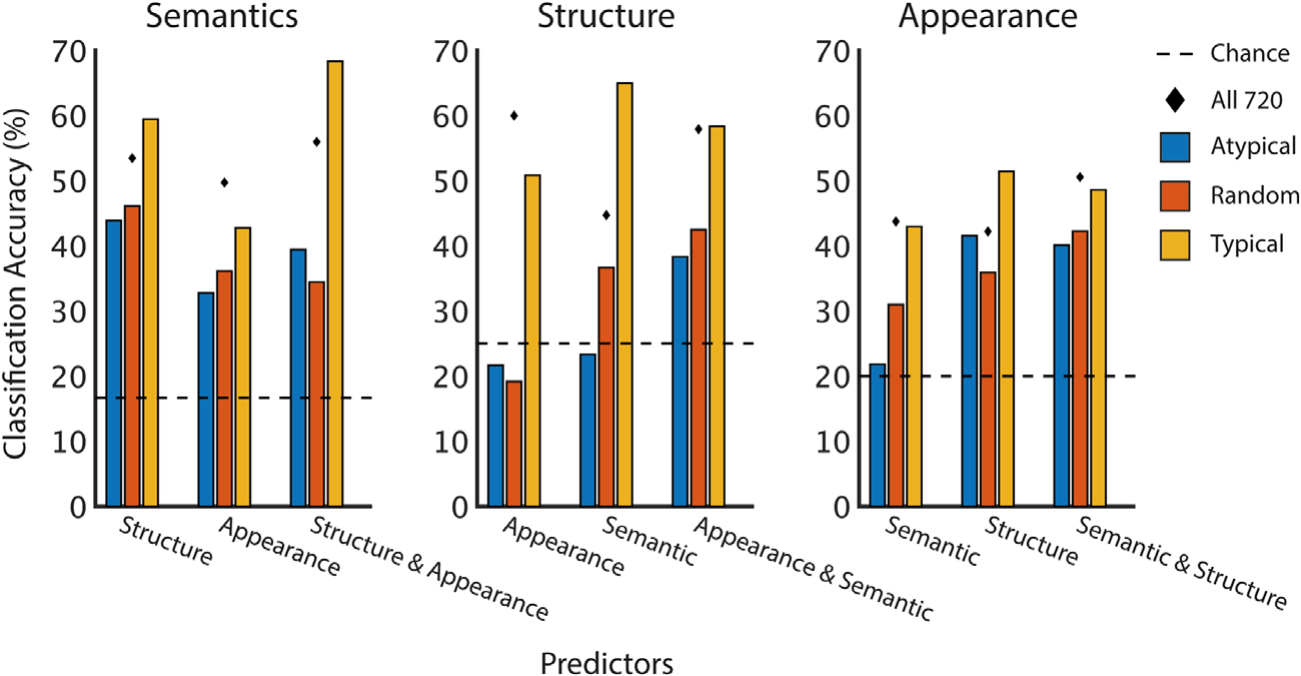
Bayes classification accuracy for random, atypical, and typical images. Most classifiers exceeded chance (1/*k*, dashed line), and typical exemplar classification was consistently more accurate than atypical and random image classification.

The typical exemplar classifier outperformed the random and atypical image classifiers in every combination of categories (see Figure 9). This typicality advantage was particularly large for semantic classification using 3D spatial structure and 2D appearance (left panel), and 3D spatial structure classification (middle panel) using semantic categories. Most of the typical exemplar classifiers also outperformed the full-dataset classifiers from Table 2, despite comparatively small training dataset sizes (120–180 vs. 720). These results confirm that relationships between dimensions are strongest for typical category exemplars.

### Discussion

In Experiment 2, we demonstrated that the categories developed from 80 images in Experiment 1 generalized well to 640 additional images, and different participants. Our category systems were not only representative of the 80 images they were derived from, they also captured the categorical structure of new images. However, given that generalization was only tested for images sampled from the same dataset, it is still possible that each category system reflects idiosyncrasies of the images used to develop them (in our case, the SYNS dataset). The SYNS scenes were randomly sampled from a diverse range of outdoor environments identified in the UKLand dataset (GeoInformation Group, www.geoinformationgroup.co.uk) to capture a wide variety of real-world scenes (Adams et al., 2016). Although we hope that this careful sampling will lead to good generalization, it remains to be seen how the category systems derived from our first experiment generalize to other image datasets.

A second question is how the categories we derived in Experiment 1 relate to existing models of scene categorization. We address this issue in our third experiment. The spatial envelope model (Oliva & Torralba, 2001) serves as a good comparison for our 3D spatial structure categories. Spatial envelope properties (e.g., Roughness and Openness) are thought to dominate early scene representations (Greene & Oliva, 2009a, 2009b, 2010) and may be encoded in a distinct cortical pathway that represents spatial boundaries (Harel, Kravitz, & Baker, 2012; Park, Brady, Greene, & Oliva, 2011). Moreover, prior work has found that spatial envelope properties predict semantic categories, but—notably—using a different set of semantic categories than those we derive from SYNS in Experiment 1 (Greene & Oliva, 2006, 2009b; Oliva & Torralba, 2001). Here we ask whether spatial envelope properties predict our SYNS-derived 3D spatial structure and semantic categories.

The relationship between low-level features that comprise an image's GIST (see Figure 2), and spatial envelope properties, may vary across datasets. Hence, we also test whether GIST features are consistently diagnostic of spatial envelope properties (regardless of the dataset), or whether this relationship is unstable and idiosyncratic. Previous computational work suggests that cluster-weighted models (CWMs) applied to GIST features are “well suited to encoding structural scene priors” (Ross & Oliva, 2010, p. 21), so we examined whether we could apply CWMs to the GIST features of SYNS images to predict human spatial envelope ratings.

To summarize, in Experiment 3 we ask human observers to directly estimate three spatial envelope properties of SYNS images (mean depth, openness and perspective). We examine how well these spatial envelope properties can be used to classify the SYNS images across the three category systems developed in Experiment 1, and assess improvements in classification as a function of typicality (as in Experiment 2). Finally, we quantify the relationship between SYNS image GIST features and spatial envelope properties, with an aim to replicate and generalize the results from Ross and Oliva (2010).

## Experiment 3

### Methods

#### Participants

Three postgraduate students, 2 male (including M.A, who was the only non-naïve participant), age range: 23–27 years, from the University of Southampton participated as volunteers. Informed consent was obtained before experimentation, and ethical approval was acquired from the Research Governance Office, University of Southampton.

#### Materials

Image and display specifications matched those reported in Experiment 2.

#### Procedure

We replicated the task performed by Ross and Oliva (2010), wherein participants viewed one monoscopic image at a time, and used three sliders to quantify the “Mean Distance,” “Openness,” and “Perspective” on a scale of 1 to 7.^3^ Participants rated all 720 images in random order.

#### Statistical analyses

First, we report human-rated spatial envelope properties across the image categories developed in Experiments 1 and 2. Second, we explore the relationship between image GIST features (see Figure 2) and human-rated spatial envelope properties. Specifically, we test whether CWMs operating on image GIST features provide a good, generalizable model of human perception of spatial layout, as suggested by Ross and Oliva (2010). Accordingly, we predicted spatial envelope properties from image GIST features using CWMs: i) trained and tested on SYNS images and ratings or ii) trained on Ross & Oliva's images and ratings, and tested on SYNS images and ratings (see Table 4). Our procedure, detailed elsewhere in this section, replicates Ross & Oliva's cross-validation method.

**Table 4.** Naïve Bayes classification accuracy per dimension (i.e., category system). Models were trained to predict each dimension from three human-rated spatial envelope dimensions, namely, openness, mean depth, and perspective.

	Subset			
	All 720	Typical	Atypical	Random
Semantic	45.76%	44.44%	37.22%	39.44%
3D spatial structure	67.73%	85.83%	64.17%	62.50%
2D appearance	39.08%	40.14%	45.77%	45.07%

By dividing an image into spatial grids of varying size (e.g., 2 × 2 or 4 × 4) and computing the GIST features at every grid location, we can obtain GIST representations with different spatial resolutions. Ross and Oliva (2010) found that the strength of the relationship between GIST features and spatial envelope properties is modulated by this spatial resolution. We therefore determined the optimal spatial resolution for (independently) predicting the three spatial envelope properties from SYNS image GIST features. First, we projected the GIST features onto the PCA bases derived by Ross and Oliva (computed from an independent, third dataset—a measure taken to facilitate model generalization). Subsequently, using five-fold cross validation, we trained CWMs to predict the human-generated spatial envelope properties from these GIST features, recording mean squared prediction errors over each left-out fold. Because CWMs are optimized for estimating data with context-dependent relationships between inputs and outputs (e.g., in our case, an enclosed forest scene may have different low-level features to an enclosed street scene; for details, see Ross & Oliva, 2010), we also cross-validated, within each spatial resolution, the optimal number of model clusters. An additional set of models, trained on Ross and Oliva's (2010) dataset and tested on the SYNS dataset, were developed to test generalization of the relationship between GIST features and spatial envelope properties (reflected in prediction accuracy relative to models trained and tested on the same dataset).

### Results

#### Human ratings

**Figure 10.**
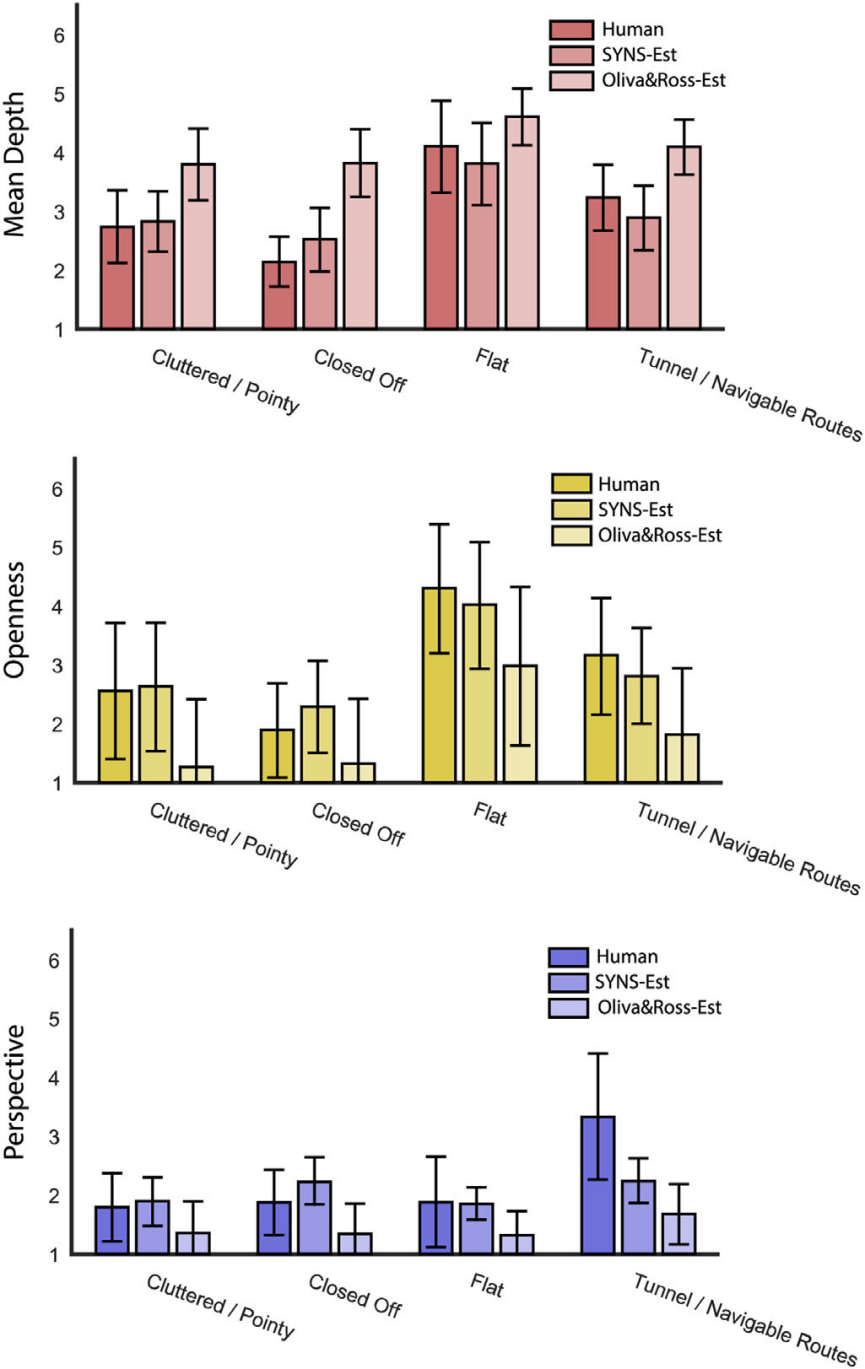
Mean human ratings (‘Human’) and CWM-estimates (‘SYNS-Est’ and ‘Oliva&Ross-Est’) for three spatial envelope properties separated by 3D spatial structure category. Error bars show ±1 standard deviation.

Figure 10 shows the human spatial envelope ratings and the CWM-estimated ratings separated by 3D spatial structure category over all 720 SYNS images. Intuitive patterns are evident in the human ratings. For example, the closed off category has low values on all three spatial envelope properties, whereas the flat category is high in openness and mean depth, and low in perspective. These results confirm that our 3D spatial structure categories capture environmental regularities also conveyed by human-rated spatial envelope properties. Indeed, a Naïve Bayes classifier trained to predict 3D spatial structure categories from the three human-rated spatial envelope properties (via LOOCV, as in Experiment 2), achieved 67.73% accuracy (Table 4)—substantially better than “prior-only” classification, predictions from semantics, 2D appearance, or both (Table 2).

Semantic and 2D appearance categories were classified from human spatial envelope ratings with 45.76% and 39.08% accuracy, respectively (see Table 4). It is worth noting that classifiers using only an image's 3D spatial structure category to predict its semantic and 2D appearance category performed better than predictions from these spatial envelope properties (Table 2).

#### Typical exemplars only

Experiment 2 demonstrated that typical category exemplars have more predictable features. Isolating the 30 images from each category with the highest interobserver agreement, and using the human-rated spatial envelope properties to predict 3D spatial structure, semantic, and 2D appearance categories, we achieved 85.83%, 44.44%, and 40.14% classification accuracy, respectively (see Table 4). Although 3D spatial structure classification showed a substantial improvement, semantic and 2D appearance categories produced negligible changes. This pattern was also found for atypical and randomly sampled images: 3D spatial structure classification was substantially poorer for atypical and random exemplars, but semantic and 2D appearance classification was relatively unaffected (Table 4).

#### CWM performance

Table 5 shows the CWM prediction errors and optimal CWM parameters for predicting human-rated spatial envelope properties from GIST, within and across datasets. CWMs learn optimal regression functions to apply for specific contexts, thereby obtaining more accurate predictions than standard linear models (Ross & Oliva, 2010). Ross and Oliva's (2010) images required higher spatial resolutions than SYNS images to optimally estimate spatial envelope properties (see Table 5). Moreover, training and testing across different datasets caused a substantial increase in prediction error (compare the SYNS/SYNS and Oliva & Ross/SYNS mean squared prediction errors in Table 5). These results suggest that the relationship between GIST features and spatial envelope properties varies between datasets. This finding cannot be attributed to weaker relationships between GIST features and spatial envelope properties, or poor suitability of CWMs for the SYNS images, because CWMs trained and tested on SYNS produce smaller errors across all three dimensions.

**Table 5.** Optimal CWM parameters and mean-squared prediction errors (MSEs) for estimating human-rated spatial envelope properties from GIST. Note: The cross-validation method for identifying the optimal CWM parameters (spatial resolution and number of clusters) for predicting the spatial envelope properties of Oliva & Ross’ images is described in Oliva & Ross (2010). The model trained on Oliva & Ross’ dataset and tested on SYNS used the optimal model parameters for the training data.

	SYNS/SYNS			Oliva & Ross/Oliva & Ross			Oliva & Ross/SYNS
Training/test data	Resolution	Clusters	MSE	Resolution	Clusters	MSE	MSE
Mean depth	1 × 1	8	0.47	4 × 4	6	0.56	1.81
Openness	4 × 4	5	0.35	8 × 8	6	0.87	1.91
Perspective	1 × 1	5	0.99	2 × 2	4	1.95	1.30

Inspection of the model-estimated spatial envelope properties across our 3D spatial structure categories in Figure 10 illustrates that the CWMs trained on SYNS data generated predictions that, for the most part, preserved category-specific patterns of spatial envelope ratings: the closed off category produced relatively low values across all three dimensions, and the flat category was low in perspective, but high in openness and mean depth (i.e., like the human ratings). By contrast, the CWMs trained on a different dataset markedly distorted these patterns: spatial envelope properties vary little between categories, and across every category, mean depth is substantially overestimated, and openness and perspective are underestimated. To explore this further, we trained two naïve Bayes classifiers to predict our 3D spatial structure categories from spatial envelope ratings i) estimated from the SYNS-trained model, and ii) estimated from the Oliva and Ross-trained model. Classification was considerably more accurate using SYNS-trained estimations (57.16% vs. 44.92%), confirming that the relationship between GIST and spatial envelope properties is unstable between datasets.

### Discussion

Human-rated spatial envelope properties are closely related to our 3D spatial structure categories. Indeed, the impressive classification performance found for typical 3D spatial structure exemplars (i.e., 85.83%) suggests that spatial envelope properties and our categorical description of spatial structure encode similar scene properties. However, the relationship between spatial envelope properties and our other category systems (semantic, 2D appearance) was weak—weaker in fact than the relationship between our 3D structure categories and those category systems.

Notably, the GIST features that predict spatial envelope properties vary between datasets, thereby impeding generalization. Although low-level differences between the datasets may account for this effect, the sensitivity of GIST to these low-level properties suggests that GIST features may not provide a robust route to scene understanding.

## Experiment 4

In a final experiment, we examine the flexibility of the CIRCA method by applying it to data collected from a larger image set, using a different experimental task. Databases like ImageNet (Deng et al., 2009), SUN (Xiao et al., 2010), and Places (Zhou et al., 2014) use semantic labels to search crowd-sourced photography sites (e.g., Google images), enabling large-scale image sampling from a wide range of environments (albeit at the expense of control over intrinsic and extrinsic camera properties). These large-scale databases are popular in computer vision and behavioral research, and the categories that organize these databases are frequently used as class labels to evaluate model/human performance (e.g., in the burgeoning field of deep learning). In Experiment 4 we test i) whether our clustering method can be applied to larger datasets and ii) how well the resultant labels capture human classification judgements, relative to the existing ground truth labels for large datasets.

Clearly, our sorting task of Experiment 1 would become infeasible for datasets containing thousands of images. However, our method can be applied to data from various experimental paradigms that produce pairwise similarity judgements. Fortunately, appropriate data already exist from a same–different experiment conducted by Greene et al. (2016).

### Method

#### Participants, materials, and procedure

Here we provide a short summary of the study conducted by Greene et al. (2016). For a complete description of the study, please refer to the original paper.

A total of 2,296 participants were recruited from Amazon Mechanical Turk (AmTurk), and stimuli were obtained by pooling 62,468 images from ImageNet (Deng et al., 2009), SUN (Xiao et al., 2010), Corel, and an additional 15-scene database (Fei-Fei & Perona, 2005; Lazebnik, Schmid, & Ponce, 2006; Oliva & Torralba, 2001).

For each trial, participants viewed two images side by side, and were asked to determine whether they belonged to the same or different category (via button press). Categories were defined by the instructions to participants: “Consider the two pictures below, and the names of the places they depict. Names should describe the type of place, rather than a specific place and should make sense in finishing the following sentence ‘I am going to the. . . .’ ” Participants also named the category of every *left* image (as a free-text response). Image pairs were selected randomly, and participants were remunerated per trial, completing as many trials as they liked.

#### Statistical analyses

To validate the CIRCA method, we compared it against two competing models.

(i) The SUN category system (Xiao et al., 2010). The majority (68.14%) of Greene et al.'s (2016) pooled dataset contains images taken from the SUN database. The SUN database was constructed by finding 2,500 unique terms in WordNet (Miller, 1995) that describe real-world environments. After collapsing over synonyms and expanding categories with multiple visual subtypes (e.g., indoor vs. outdoor views of churches), 899 category labels emerged, and images for each category were retrieved by downloading the images returned by various search engines (e.g., Google Images).
(ii) Greene's (2019) clustering method. Greene (2019) proposed a simple clustering method using the same–different judgements in the dataset described above (Greene et al., 2016). First, images are assigned to their respective SUN, ImageNet, and Corel categories, and the proportion of trials in which observers responded “same” is computed for images from the same category, and images from different categories. This process is completed for every pair of categories to build a by-category similarity matrix. Categories (and the corresponding images) that produce *within*-category similarities of less than 0.75 are removed. Pairs of categories that produce *between-*category similarities of greater than 0.5 are merged. Note that this method removes and merges whole categories, and does not operate on individual images.

For a fair comparison with both of these models, we only retained the 42,927 images retrieved from the SUN database, used by Greene et al. (2016). Of the approximately 921 million possible pairwise combinations of these 42,927 images, approximately 2.5 million (0.27%) were presented to participants at least once. Because the vast majority of image pairs never occurred in this experiment, the resulting 42,927 × 42,927 similarity matrix is highly sparse. Missing data introduces uncertainty: two images without similarity data could belong to the same or different categories. To minimize sparsity, we used an iterative sampling procedure to find the most densely connected subset of images (i.e., with the largest number of observations). We first selected the single image with the largest number of unique pairings with other images. Further images were iteratively added to our sample, by finding, on each iteration, the image with the maximum number of connections (i.e., same–different judgements) with the images already in the sample. For the current study, we selected the 1,000 maximally connected images. Of the 499,500 possible unique pairings, 31,884 (6.38%—a vast improvement over 0.27%) had similarity data in this sample.

In Experiment 1, we protected against overfitting while finding the optimal number of clusters by testing model predictions against data produced by left-out participants (i.e., leave-one-out cross-validation). As the current dataset omits participant identifiers, we used k-fold cross-validation on individual *trials* instead. In most other respects, we simply replicated the analyses described in Experiment 1. In short, we determined the optimal number of clusters by splitting the trial-by-trial data (i.e., individual similarity judgements) into 10 equally sized folds, training on nine folds, and testing on each left-out fold in turn.

### Results

**Figure 11.**
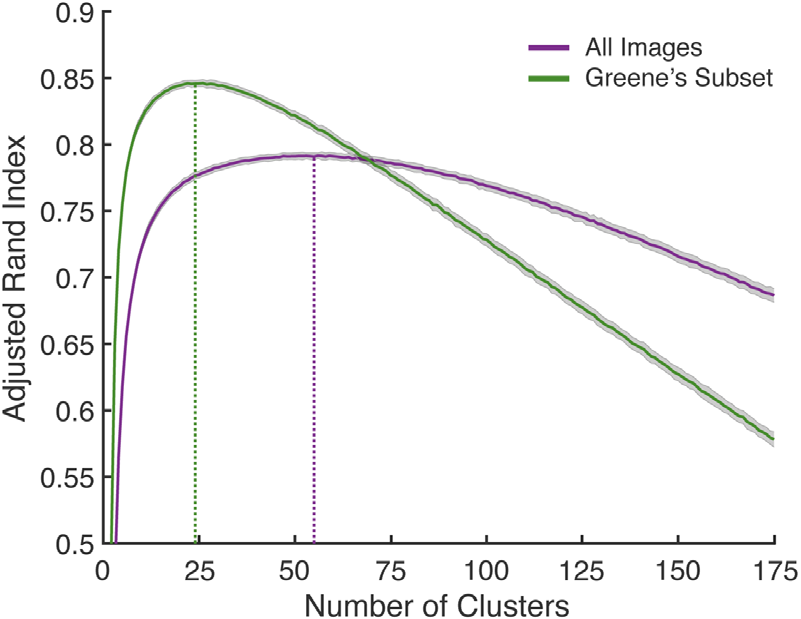
The optimal number of categories was determined via 10-fold cross-validation. The *y*-axis gives the ARI averaged over 10 folds, and 1,000 different random initializations of the CIRCA method per fold. Shaded areas around each line represents ±1 standard deviation. The optimal number of categories (vertical dashed lines) is 55 for all 1,000 images, and 24 for Greene's subset.

Figure 11 (purple line) shows the resulting ARI using our 10-fold cross validation over the 1,000 selected images, as a function of the number of clusters. The curve has been smoothed by kernel regression, with kernel scale optimized by leave-one-out cross-validation on the mean ARIs. We find that the ARI peaks at 55 clusters, somewhat less than the 72 SUN categories present in our sample of 1,000 images.

After fixing the number of clusters to the optimal number, the CIRCA method was rerun with all of the data included from 10,000 random initializations, to find the clustering that maximized the ARI. The resulting ARI is slightly higher than the ARI produced by the SUN category system (see Table 6, rows 1–2). When we (suboptimally) increased the number of categories to 72, to match the number of represented SUN categories, the ARI decreased as expected, but remained favorable compared with the SUN system (Table 6, row 3).

**Table 6.** The ARI evaluates how well each model predicts similarity judgements in the same–different task. For the sample of 712 and 1,000 images, the CIRCA method outperforms the two alternative models: the SUN category system, and Greene's (2019) clustering method.

Model	No. of images	No. of categories	ARI
CIRCA	1,000	55	0.7703
SUN	1,000	72	0.7354
CIRCA	1,000	72	0.7697
CIRCA	712	24	0.8331
Greene	712	22	0.8196
CIRCA	712	22	0.8328
SUN	712	35	0.8326
CIRCA	712	35	0.8338

Greene's (2019) method removes 26.17% of trials by excluding categories with an average within-category similarity rating of less than 0.75, leaving 712 images organized into 22 categories (merging 35 SUN categories). For a fair comparison against Greene's method, we repeated the CIRCA method with this reduced sample of 712 images. Cross-validation revealed that 24 clusters was optimal for this subset (Figure 11, green line) and the resulting optimal clustering ARI compares favorably with Greene's categories and the SUN categories (Table 6, rows 4–6). Finally, we compared these alternative models to our method for this image subset when we matched the number of clusters (Table 6, rows 7–8). Our method outperformed the two competing models, regardless of sample size, and regardless of whether we used the optimal number of clusters, or simply matched the number of clusters.

**Figure 12.**
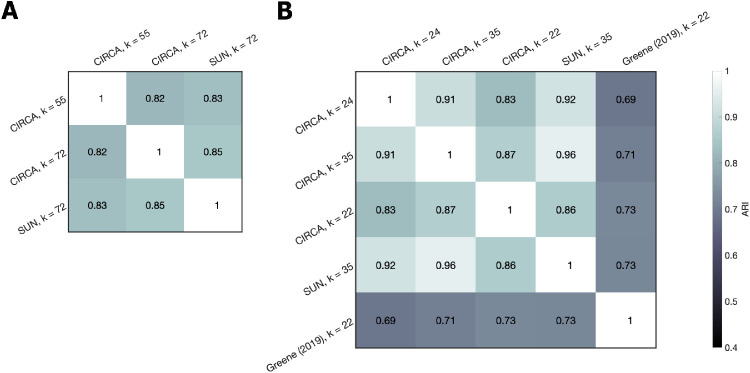
Cluster similarity between models quantified using the ARI. (A) In the 1,000-image subset, our model produced clusterings that were highly similar to the SUN model. (B) In the 712-image subset, we observed similarly high agreement with the SUN clusterings, particularly when we matched the number of clusters. Greene's method produces markedly different clusterings.

We can also examine the similarity of the clusterings produced by the different methods using the ARI. Our method produced clusterings highly similar to the SUN model, whereas Greene's method produced clusterings that differed from the other two (see Figure 12). When the number of clusters was matched, there was very close agreement between our method, derived using human same-different judgements, and the SUN system, derived entirely independently, via label-driven image searches (ARI = 0.96, Figure 12B).

**Figure 13.**
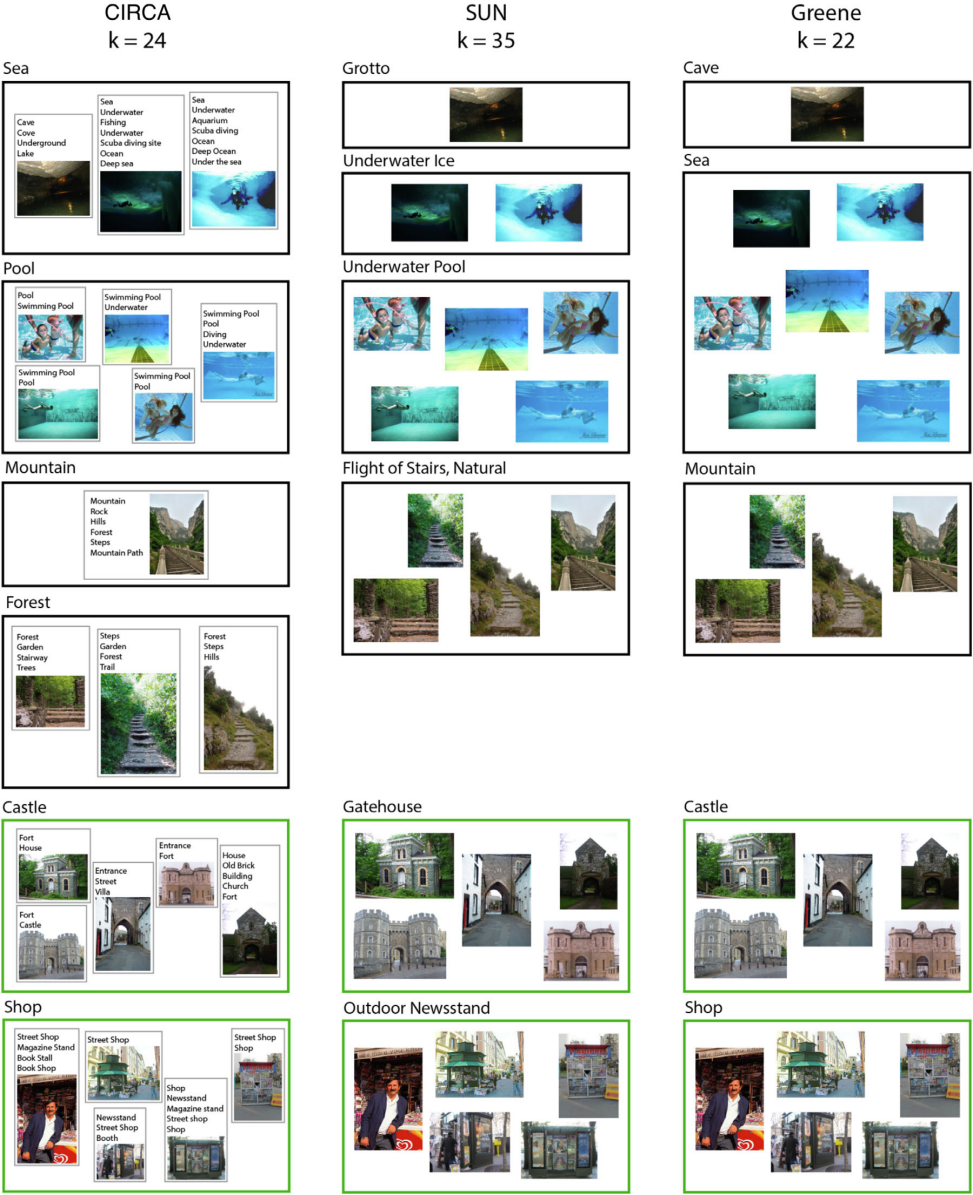
Example categories produced by our clustering method (left column), the SUN system (middle column), and Greene's method (right column). To generate these examples, we sampled a small subset of images used in Experiment 3, and assigned them to their respective categories according to the three different category systems. Bounding boxes show the different categories, and labels above each box are the category labels either retrieved from the SUN database (middle column), or derived by computing the mean word-vector of the participant-generated labels. In the left column, we provide a small number of example labels assigned to the images by observers. Green bounding boxes (bottom) signify that all three models generated the same category.

Examples of agreements and disagreement between the three models are illustrated in Figure 13. While the SUN system separates ‘Grotto’ and ‘Underwater Ice’ images, our category system combines both into a “Sea” category (our labelling method is described elsewhere in this article). Also, our method splits “Flight of Stairs, Natural” into “Mountain” and “Forest” categories based on the global context in which the stairs occur. Greene's method subsumes “Underwater Ice” and “Underwater Pool” under a single “Sea” category. Importantly, however, many categories are identical across all three models (green bounding boxes).

Another way of comparing our clustering method to existing models is to analyse/analyze the category labels that observers assigned to every left image in Greene's experiment. A good category system should maximize the variance in word meaning between category labels, and should minimize variance within categories. Put simply, categories should represent independent concepts, but members of the same category should be relatively homogenous. We quantify word meaning using Word2Vec (Mikolov, Sutskever, Chen, Corrado, & Dean, 2013). Word2Vec is a family of shallow, two-layer neural networks that produce word embeddings. These models are trained to predict the identity of single words from neighboring words taken from the same sentence (using large-scale corpora). The hidden layer varies in dimensionality (from 100 to 1,000; Mikolov, Chen, Corrado, & Dean, 2013) and represents a vector space. Each unique word inputted during training is assigned a corresponding “word vector,” or embedding, in this vector space. A useful property of these embeddings is that they are organized semantically, and can be manipulated algebraically (a well-established example is *king* − *man* + *woman* = *queen*). For our purposes, Word2Vec offers a useful quantitative representation of word meaning to evaluate clusterings.

We used the GloVe model, which represents each label as a 300-dimensional word vector (Pennington, Socher, & Manning, 2014), to derive word embeddings for all the participant-generated labels. Word vectors were converted to unit vectors, and organized into categories based on the category of the images they describe. We calculated the grand-mean word-vector, and, for each category system, the category-mean word-vectors. Then, we computed i) the (summed) squared Euclidean distance between the grand-mean and the category-means (D_*between*_) and ii) the (summed) squared Euclidean distance between the category-means, and the individual word-vectors within categories (D_*within*_). These two distances are the same as to the between-group and within-group variance estimates in the standard *F*-test. Accordingly, we tested model fit by calculating the ratio between D_*between*_ and D_*within*_:

(5) $$F=\;\frac{D_{between\;}/\;df_{1}}{D_{within}\;/\;df_{2}}$$

where *df*_1_ and *df*_2_ are the degrees of freedom: *df*_1_ = *k* − 1, and *df*_2_ = *N* − *k*, where *N* represents the number of observations or labels, and *k* represents the number of categories.

Our clustering method produces higher *F*-ratios when we use the optimal (cross-validated) number of clusters, or match the number of clusterings in Greene's method (see Table 7). However, when we match the number of clusters in SUN, the SUN model achieves a higher *F*-ratio.

**Table 7.** The F-ratio quantifies the variance in word meaning captured by the models. Our CIRCA method produced a superior fit to labelling data when we used the optimal number of clusters (24 and 55), but performed worse when we matched the number of clusters in SUN.

Model	No. of Images	K categories	F
CIRCA	1,000	55	537.22
SUN	1,000	72	447.36
CIRCA	1,000	72	417.68
CIRCA	712	24	1127.15
Greene	712	22	1121.06
CIRCA	712	22	1190.57
SUN	712	35	815.49
CIRCA	712	35	794.52

Using the Word2Vec representation, we can derive single-word category terms by computing the centroid (i.e., mean) word-vector for each category.^4^ Examples of category terms produced using this method, alongside the raw participant-generated labels, are presented in Figure 13 (left and right columns, above each box/category). Compared with the category terms used in the SUN database, these terms are more general. For example, for the SUN category “Gatehouse,” human participants preferred to use the more general term “Castle” (gatehouses are typically in the same grounds as castles). This process of simplification is similarly borne out in the clustering results: our method produces fewer clusters than the SUN category system.

### Discussion

In Experiment 4, we investigated the scalability of our proposed clustering algorithm. We applied our method to human data from a large-scale same–different experiment, and tested our clusterings against two alternative models: the SUN taxonomy, and a simple thresholding method proposed by Greene (2019). We found that our method outperformed both models. Moreover, we tested whether our category system was more consistent with the labels used by participants in the same experiment. When we used the optimal number of clusters—determined via cross-validation—our clusterings outperformed the SUN category system. These results suggest that our method can be applied to data from different tasks, and larger datasets.

## General discussion

We proposed a behaviorally grounded method of deriving category systems for real-world scenes, and validated it on the SYNS (Adams et al., 2016), and SUN databases (Xiao et al., 2010). In Experiment 1, we instructed participants to categorize 80 SYNS images by their i) semantic content, ii) 3D spatial structure, and iii) 2D appearance, in a free-sorting task. We determined the optimal category structure for each task and assigned participant-generated labels to each category.

In Experiment 2, a separate set of participants used the optimal labels from Experiment 1 to categorize a larger set of 720 SYNS images. We produced strong evidence that our category systems generalized over a larger set of images. Moreover, we found stable category associations that enabled predictions of category membership in one dimension from categorical properties across other dimensions.

In Experiment 3, we labelled the SYNS dataset using three spatial envelope properties and found a reliable relationship with the 3D spatial structure categories, and weaker relationships with the semantic and 2D appearance categories. We showed that without dataset-specific training, GIST features are not diagnostic of spatial envelope properties or scene category.

In Experiment 4, we tested our method on data from a same-different task using 712 to 1,000 images from the SUN database. Our method generated categories that predict same/different judgements more accurately than the SUN taxonomy, and an alternative clustering method (Greene et al., 2016). Moreover, our method generated categories that captured a greater amount of variance in the meaning of participant-generated labels.

### Deriving participant-driven category systems

Image categorization is a popular metric for scene recognition, yet potential problems with *contrived* categorical taxonomies of real-world scenes are seldom discussed. In most categorization research, participants are presented with category labels that ostensibly represent the ground-truth categorical structure of real-world environments. However, different studies use different category systems, under an implicit assumption that variations in categorical structures have little or no effect on participant behavior. Surprisingly, this assumption is maintained despite the known inequality of different categorical descriptions (e.g., between basic-level and superordinate category systems; Sofer et al., 2015). We have argued that different category systems codify different visual features and thus experimental categorization tasks will produce unnatural behavior insofar as applied category systems fail to reflect human-preferred taxonomies of real-world environments. Our participant-driven method of deriving category systems directly identifies these human-preferred taxonomies and thereby provides a means of obtaining a more principled ground-truth.

### Properties of the SYNS category systems

Applying our method to the semantic categorization task in Experiment 1 generated intuitive labels like “road,” “car park,” “residence,” and “beach”—all of which resemble commonly applied categories in past research (e.g., “highway,” “coast,” etc.; Fei-Fei et al., 2007; Fei-Fei & Perona, 2005; Oliva & Torralba, 2001). Interestingly, however, most existing category systems discriminate between forest and countryside categories. Forests and countryside are basic-level members of the superordinate nature category; existing scene taxonomies assume a sharp division between these two levels of representation, partitioning categories into discrete multilevel hierarchies (Rosch & Lloyd, 1978; Tversky & Hemenway, 1983). Our “nature” category unifies forest and countryside scenes, thereby intermingling superordinate and basic basic-level categories. This finding suggests that the accepted demarcation between superordinate and basic-level scene categories may be fuzzier than previously thought. Although it is also possible that the “nature” category was produced by averaging over two types of participant, namely, i) those that generated superordinate categories, and ii) those that generated basic-level categories, we introduced a constraint on the number of categories to prevent this problem. Within the specified range of three to 10 categories, the minimum number of semantic categories used by any participant was five. Hence, it is doubtful that some participants were just performing superordinate categorization.

The variability in the granularity of individual categories within a category system can be interpreted as an extension of what Rosch and Lloyd (1978) described as the economic balance between low cognitive effort and maximum discriminability (although they asserted that this was limited to basic-level category systems). Representing some categories coarsely, and other categories at a finer level, may be optimal under certain conditions. For example, plants and animals are hierarchically classified according to species, genus, family, and so on, in Western scientific taxonomies. Many *non*-Western cultures share similar taxonomies, but eschew some redundant distinctions in favor of more generic categories that have greater cultural utility (thereby generating sets of categories with mixed granularity; for a review, see Malt, 1995). Real-world scene categories may vary with similar observer characteristics, such as stimulus familiarity, motivation, expertise, and of course, culture, that cause humans to use mixtures of coarse and fine distinctions.

Our 3D spatial structure categories strongly resemble Oliva and Torralba's (2001) spatial envelope properties. Categories of “flat” and “closed off” seem to correspond to opposing poles along the openness dimension, “cluttered” or “pointy” corresponds with “roughness,” and “tunnel” or “navigable routes” resembles the “expansion/navigability” dimensions (Greene & Oliva, 2006; Oliva & Torralba, 2001). In Experiment 3, we verified this mapping by testing the performance of Bayes classifiers trained to predict 3D spatial structure category by encoding variations in human-rated spatial envelope properties. We found that spatial envelope properties were strong predictors of category membership, achieving 85.83% classification accuracy for typical category exemplars. The convergence of our 3D spatial structure categorical model and the spatial envelope model (Oliva & Torralba, 2001, 2006) suggests that both capture a robust vocabulary of natural scene statistics.

A key tenet of the spatial envelope model is that humans compute an intermediate representation of 3D spatial structure, which is in turn used to infer semantic category during early visual processing (Greene & Oliva, 2006, 2009a, 2009b, 2010; Oliva, 2005; Oliva & Torralba, 2001, 2006; Torralba & Oliva, 2002). In support of this model, previous work has shown that scene structure is extracted from natural scenes before semantic categories are accessed (Greene & Oliva, 2009a), and that humans use spatial structure cues to inform judgements of semantic category (Greene & Oliva, 2009b, 2010). Although we did not manipulate presentation duration directly, in Experiment 2, we did find that a classifier trained to predict semantic category from 3D spatial structure category produced reasonable results: 57.36% accuracy on all images, and 68.33% accuracy on only the typical category exemplars. However, in Experiment 3, we also found that, human-rated spatial envelope properties are poor predictors of semantic categories (45.76% correct). The reduced discriminative power of spatial envelope properties (compared with our spatial structure categories, and other classification results; e.g., Greene & Oliva, 2009b) may be due to the taxonomical structure of our empirically derived semantic category system. Perhaps the impressive performance of previously reported spatial envelope-driven semantic classification (e.g., Greene & Oliva, 2009b) is produced, in part, by the selection of semantic categories that are discriminable based on spatial envelope profiles (i.e., spectral signatures; see Oliva & Torralba, 2001). It is also possible that this result is caused by an idiosyncratic set of SYNS categories. Future research should examine whether empirically derived category systems from other datasets also produce a weak association between spatial envelope properties and semantic content. Or, perhaps a simpler explanation exists: prior studies testing semantic classification from spatial envelope properties have used up to seven properties (Greene & Oliva, 2006, 2009b), while we used three (used by Ross & Oliva, 2010). We would likely see an improvement in classification performance if we used additional properties like “navigability” and “temperature” (Greene & Oliva, 2006, 2009b).

Although observers were instructed to sort images based on multiple, complex, feature dimensions, including “patterns,” “textures,” and “color,” our 2D appearance categories contain only two distinguishable feature dimensions: color (blue, green, brown) and global luminance (bright and dark). Although color is known to be informative for scene understanding (Castelhano & Henderson, 2008; Goffaux et al., 2005; Goffaux et al., 2003; Oliva & Schyns, 2000), no prior studies have investigated the importance of global luminance properties. Furthermore, no efforts have been focused on formulating chromatic/luminance categories for real-world scenes (although Oliva & Schyns [2000] did use color histograms to examine the diagnosticity of color between different *semantic* categories).

The open endedness (i.e., multidimensionality) of the 2D appearance task instructions may explain the greater disagreement between observers relative to the semantic task (Figure 4), although it does not explain why agreement was higher than for the 3D spatial structure task (which was more constrained). Either way, it is entirely possible that different observers were grouping images based on different feature dimensions—a problem that highlights the importance of carefully designing and standardizing observer instructions.

Various characteristics of the sorting task in Experiment 1 may undesirably bias human behavior away from natural categorization. The task instructions, number of images, constraints on the number of images allowed per category (more than one), and the range of permitted categories (three to 10) may influence observer sorting patterns. While it is difficult to conduct a categorization experiment with *no* constraints on behavior, it would be beneficial for future work to investigate how various task demands bias categorization.

### Estimating spatial envelope properties using cluster weighted models

Ross and Oliva (2010) previously suggested that CWMs are “well suited to encoding structural scene priors” (pp. 21). Yet, in Experiment 3, we showed that the relationship between low-level GIST features and spatial envelope properties—a relationship encoded by the proposed CWMs—varied with the chosen dataset. We demonstrated that models trained on Ross & Oliva's dataset produce inaccurate estimations of spatial envelope properties in the SYNS dataset. Similarly, the optimal spatial resolutions for estimating spatial envelope properties varied between the datasets. Although the cause of this dataset-dependency is unclear, it is conceivable that the perception of mean depth, openness and perspective co-vary with the photographic field of view, that is, the focal length of the camera, which will determine the amount of perspective apparent in the image. While the field of view of the stereoscopic SYNS images we used was fixed at 31.12 × 22.36° (Adams et al., 2016), the spatial envelope literature is based on crowd-sourced photography—images taken from multiple different cameras, presumably with varying focal lengths. Spatial perception may also depend upon camera pose. The SYNS stereo pairs were all taken at eye height, with a horizontal optical axis. In contrast, the crowd-sourced images used for spatial envelope work vary substantially in camera height and angle. The sensitivity of the GIST representation to low-level differences caused by camera properties, or even simpler changes like modifications to global contrast (which also affects the GIST [Oliva & Torralba, 2001], but has no effect on the spatial structure of an image), suggest that they may be poor at representing scene layout information.

GIST is a popular low-level summary statistic in computer vision, yet recent advances in convolutional neural networks (CNNs) has produced better models of spatial structure processing. For example, Cichy et al. (2017) measured the correlation between human MEG responses to the dimension of scene size (i.e., the expansiveness of a scene), and the predictions of three competing models: GIST, HMAX (a biologically inspired hierarchical model; Serre, Wolf, & Poggio, 2005), and a CNN trained to classify scenes from the Places database. The CNN produced layer activations that correlated more strongly with human responses than the other two models. It is plausible then, that, with a state-of-the-art model of spatial structure estimation (e.g., an appropriately trained CNN), we might observe less dataset dependency and stronger predictions of spatial structure properties. A thorough analysis of how CNN feature representations relate to spatial structure categories is beyond the scope of this paper, but future research may address this problem.

### Typicality enhances category discrimination

Some images or scenes are clearer category members than others. Prototype theory conceptualizes category membership as the proximity of an instance to a central exemplar (Rosch, 1999; Rosch & Mervis, 1975). Typical category instances have the “most attributes in common with other members of the category and [the] least attributes in common with other categories” (p. 573; Rosch & Mervis, 1975). Real-world scene categorization behavior supports this theory: Torralbo et al., (2013) found that the variance in spatial structure and color for typical images is smaller than atypical images. Typical category exemplars are categorized more efficiently than atypical images (Torralbo et al., 2013), and classifiers trained on global image features achieve greater accuracy for typical category exemplars (Ehinger et al., 2011). Our findings are consistent with these results. In Experiments 2 and 3, we demonstrated that typical category exemplars are easier to classify than atypical or randomly sampled images.

### The scalability of our clustering method

The categories derived from the SYNS database may not be suitable for application to all other databases. Large-scale image repositories such as Places (Zhou et al., 2014), ImageNet (Deng et al., 2009), and SUN (Xiao et al., 2010) have a greater range of environments than SYNS, and while the SYNS dataset was designed to maximize environmental variation, some scenes such as deserts and mountains—that would conceivably comprise independent categories—are not included, as they do not occur in the sampled region of southern England (Adams et al., 2016). In Experiment 2, we tested the generalizability of our category systems on novel images taken from the same locations and using the same camera, with the same focal length, and so on. A stronger test of generalization might draw data from additional image repositories, but this introduces the problem of applying unsuitable taxonomies to new and different datasets.

To circumvent this problem, in Experiment 4, we applied our method to a distinct dataset used in a same–different psychophysical task, in which participants viewed pairs of images sampled from the SUN database and i) judged whether they were drawn from the same or a different semantic category and ii) typed a category label for the left image (Greene et al., 2016). We found that the categories generated by our method outperformed the SUN taxonomy, and a competing clustering method (Greene, 2019), in predicting human same/different judgements, and in capturing variance in the meaning of participant-generated image labels.

It is worth noting, however, that performance differences were sometimes minor (see Tables 6 and 7). In fact, we observed a strikingly high agreement (near-perfect, when the number of clusters is matched) between the clusters generated by our method, and the SUN taxonomy. This result is impressive because the SUN taxonomy was developed completely independently of the experimental data used to derive our categories. The SUN categories were determined by identifying place names represented in WordNet, collapsing over synonyms, and then using these as search-terms in various search engines to retrieve images (Xiao et al., 2010).

WordNet organizes words into concepts by grouping synonyms into sets termed synsets. These synsets are structured hierarchically—a design decision inspired by early investigations of semantic memory (Collins & Loftus, 1975; Miller, 1990). Expert lexicographers generated these synsets *manually*. Consequently, the similarity between the category systems produced by the “WordNet approach” and our data-driven approach, may reflect the universality of how category systems are represented by humans, lexicographers and psychology participants alike. Moreover, these results suggest that linguistic taxonomies generalize to *visual* scenes—a finding consistent with research showing that long-term semantic memory is modality independent (Coccia, Bartolini, Luzzi, Provinciali, & Lambon Ralph, 2004; Simanova, Hagoort, Oostenveld, & Van Gerven, 2014).

One prominent difference between the SUN taxonomy and our category systems is the number of categories. Using *k*-fold cross-validation, we found that the optimal number of categories for 1,000 and 712 images was 35 and 24, respectively. The SUN taxonomy has more than twice this number (72 and 55 categories for 1,000 and 712 images, respectively). Our method generated simpler category systems with a larger number of images per category. This difference may reflect the fine-grained differentiation between different word meanings in WordNet. Humans show substantial disagreement regarding the meaning denoted by different WordNet Synsets (Chklovski & Mihalcea, 2003), and, in the NLP literature, merging synsets into simpler taxonomies improves word-sense disambiguation (Navigli, 2006; Snow et al., 2007).

In terms of human behavior, the preference for coarse-grained taxonomies may relate to basic-level categorization. Humans show a reliable bias toward categorizing stimuli (visual and nonvisual) at the basic level (Hajibayova, 2013; Rosch, 1999; Rosch & Lloyd, 1978; Rosch & Mervis, 1975; Rosch, Mervis, Gray, Johnson, & Boyesbraem, 1976; Tversky & Hemenway, 1983), and previous work has demonstrated that visual scenes involuntarily activate basic-level semantic concepts (Greene & Li, 2014). An inspection of the category labels assigned to our categories supports this explanation: the SUN gatehouse category is labelled “castle” and the outdoor newsstand is labelled “shop” (see Figure 13). Because gatehouses are typically enclosed within the grounds of castles and outdoor newsstands are a subtype of shop, participants seem to be collapsing over more fine-grained categories. Similarly, the SYNS semantic category system derived in Experiment 1 is mostly comprised of basic level-categories, with the exception of the superordinate “Nature” category. Taken together, our findings suggest that humans represent large numbers of visual scenes using a relatively small set of coarse-grained categories.

Greene's (2019) category systems showed weaker agreement with the SUN taxonomy and the categories generated by our method (see Figure 12). This result may relate to the constraints within Greene's clustering method: SUN categories can be eliminated or merged, but new categories cannot be created by dividing SUN categories into smaller units. For example, our method produced two separate categories: “Forest” and “Mountain” for the single SUN category: “Flight of Stairs, Natural,” based on the environmental context. By contrast, Greene's method simply reproduced the original SUN category (see Figure 13). Despite these differences, both clustering methods frequently produced identical categories (Figure 13, green boxes), and can be used for different purposes: our method can be used to derive clusterings in the absence of any assumptions about the taxonomical structure of the dataset; Greene's (2019) method can be applied as an inexpensive method of simplifying and refining existing category systems.

### The limits of our clustering method

Our method produced reasonable clusterings for a sparse dataset (see Figure 13) in which more than 90% of the datapoints were missing. Moreover, as reported in the Supplementary Materials, we tested our clustering method on simulated data, and compare the results against two alternative methods (*k*-medoids and spectral clustering). We found that our method was more robust against high levels (50%) of response noise. We also tested the behavior of our method under conditions of high interparticipant disagreement, and found that our method produced the correct number of clusters even when interparticipant disagreement was as high as 25%. Thus, our method can be safely applied to experimental data: i) containing a large amount of missing data, ii) with high levels of response noise, and iii) collected from a heterogenous population, where interparticipant agreement may be low.

We tested our method on 80, 712, and 1,000 images, but many large-scale databases contain hundreds of thousands or even millions of images. The sorting task in Experiment 1 works well for a small number of images (in our case, 80), but with larger sets of images, the workspace would quickly become cluttered and unmanageable. A physical sorting task, where participants arrange pictures of scenes in a large, open space, might fare better, but this comes with its own limitations (e.g., error-prone manual data entry, time consuming to run).

The same–different task described in Experiment 4 may seem better, but the number of judgements needed to “fill” a similarity/confusion matrix increases quadratically with the number of images—a fact highlighted by Greene et al. (2016), who recruited more than 2000 participants, and only managed to collect data for 0.27% of the possible image combinations (of 42,000 images).

Assuming a full, large-scale dataset can be practically collected, an additional limiting factor is computational efficiency. Because the number of possible clusterings given *n* stimuli and *k* clusters is *k^n^*, the search space grows rapidly as the dataset size increases. In the Supplementary Materials, we examine the efficiency of our method as a function of the number of stimuli (*n*), and number of clusters (*k*), and show that, while runtime increases with both these variables, our method is still computationally feasible for large datasets (albeit slow when *n* and *k* are large, for example, *n* = 50,000, *k* = 500).

Our method is also not limited to the domain of scene categorization: it can be applied to data collected from any psychophysical experiment that yields similarity judgements between pairs of stimuli. For example, our method could be used to derive object, color, and texture categories, and could be applied to other modalities to investigate auditory, tactile, and olfactory processing.

### Further questions regarding the utility of categories

Although it is evident that humans use categorical descriptions in everyday life to communicate notions of *place* or *location* using labels like “Beach” and “Residential,” categorical representations do not capture intracategory variations. Further, we have assumed that any given image must belong to exactly one category within a category system, whereas it may be more natural to allow images to belong to multiple categories (Patterson, Xu, Su, & Hays, 2014). For example, a scene of a house on the seashore may belong to both “Beach” and “Residential” categories. In contrast, attributes (e.g., materials or functions) can traverse category boundaries and capture intracategory variation. Attribute perception may complement category representations by providing the fine-grained information that categories lack (Ferrari & Zisserman, 2008).

Nativist approaches to category systems posit that categorization behavior reflects a universal taxa of perceptual ordering (Berlin & Kay, 1991; Rosch & Lloyd, 1978). Other investigators have stressed that labelling systems vary to a large extent across individuals and cultures (Hajibayova, 2013; Levelt, 2014). For example, highly familiar category instances (e.g., to a Neapolitan, Mt Vesuvius may be a familiar instance of “mountain”) are accessed at the individual, rather than the categorical level (Anaki & Bentin, 2009). Personal expertise may therefore determine whether a scene is identified categorically or not. This factor casts doubt over the generality of not only our categorization system, but fixed categorical taxonomies in general. Future research will benefit from assessing how individual, geographical and cultural variables shape psychological category representations (Nisbett & Masuda, 2006). Our category formation method could serve as a useful tool for investigating these problems.

## Conclusion

Scene understanding is commonly measured by assessing categorization behavior, but these measurements will only be useful if the right category system is used. We have proposed a novel method for generating participant-driven category systems. Using stereoscopic images of real-world scenes from the SYNS database (Adams et al., 2016), we established ground-truth categories across three dimensions (semantics, 3D spatial structure, 2D appearance). We explored some basic characteristics of our categories, and presented results that suggest color and spatial structure provide intermediate representations useful for determining semantic category. We then tested our method on a larger dataset, and observed a superior agreement with human judgements than rival category systems, but also a surprising degree of agreement between our clusterings, and the categories represented in the SUN taxonomy. Further simulations revealed that our method is robust against response noise and participant heterogeneity. This method may be useful for creating and/or evaluating class label systems for existing databases and for investigating specific hypotheses regarding the organization of categorical constructs.

## Supplementary Material

Supplement 1

## Footnotes

1 The fact that power spectra can be encoded efficiently, and discriminate semantic categories well, does not mean they are necessarily used by humans. Indeed, humans process the presence or absence of animals in natural scenes without exploiting the information available from the power spectrum (Wichmann, Drewes, Rosas, & Gegenfurtner, 2010).

2 We use the standard Rand index for coordinate ascent, and the ARI for cross-validation because, for ***S*****,** the number of categories is undefined and has to be approximated by ***c***. For ***c*** and the categories generated by the left-out subject, the number of categories is defined, so ARI is the preferred metric.

3
Ross and Oliva (2010) additionally instructed subjects to perform a natural vs. man-made categorization, but, because the semantic category system from Experiment 1 challenges the assumption of a sharp division between superordinate categories and finer categorical representations, we omitted this judgement.

4 In Experiment 1, participants assigned labels to whole categories, whereas in Experiment 4 they were assigned to single images. Therefore, we cannot use the same method as in Experiment 1.

## Appendix

### Participant instructions

You will view a large number of images from different scenes. Your task is to organize them into groups by using the mouse to drag and drop the images. You are free to organize the images into between three and 10 groups. All groups should contain more than one image, but they do not have to all match in size.

### How to Group

#### Semantic

Your task is to group the images according to *type of place*. Think about the *themes* across the images. Put together images that share a common place category. One possible example is the category of ‘mountains.” In this case, all mountain images would be placed in a single group.

#### Three-dimensional spatial structure

Your task is to group together images that share similar 3D properties. Think about the structure of the real scene depicted in each image. Consider the model that you would have to physically build to represent each scene. For example, you might decide that some scenes are made of one uniform surface—the ground plane.

#### Two-dimensional image appearance

Your task is to group together images that contain similarities in their 2D appearance. For example, you might think commonalities in the colors, patterns, or textures.

It is key to remember that there are no right or wrong judgements—choose whichever image combinations make sense to you. Try not to focus on particular objects within the images. For example, do not group images according to whether or not they contain a car or a person. However, it is possible that your groups or labels may be influenced by the type of objects within the scenes.

To group multiple images together, all you need to do is overlap them in the central workspace in front of you. Press down the mouse wheel to reverse the order of the images, revealing the hidden ones that have other images stacked on top of them. To get a better, 3D view of any image, select it with the left mouse button and simultaneously press down the mouse wheel. We encourage you to view the larger, 3D images when making your grouping judgements. When you have finished moving the images around and you are happy with your groups, you can press the ‘GROUP’ tab on the bottom of the display. You will see that each image within a group will be shown with the same color frame. This is to help you catch any sorting errors. If any of your groups have a wide, black frame around them, this means that you have created either i) too many categories, ii) too few categories, or iii) not enough images in one or more categories. Please return to the ‘SORT’ stage if you see any black frames. You can only manipulate the images when the ‘SORT’ tab on the bottom of the display is highlighted. Finally, assign each of the categories a label/set of labels. You are limited to one to five labels for each group, and each group *must* have a corresponding label. Once this is done, you have completed the task.
