# Supplementary material for: Category systems for real-world scenes
Source: J Vis. 2021 Feb 17;21(2):8. doi: 10.1167/jov.21.2.8 (PMC7900870; doi:10.1167/jov.21.2.8)
Supplement: Supplement 1 [file jovi-21-2-8_s001.pdf]

Supplementary Materials 1

Here we offer a brief overview of time complexity of the CIRCA algorithm. To examine how time-to-convergence is affected by the number of images, n and the number of categories, k, we simulated data from noisy observers, and varied n from 50 to 500, and k from 2 to 40. We averaged over 20 starting positions for every combination of n and k. Runtime was tested using a MATLAB R2019B implementation of the CIRCA algorithm (*https://github.com/mattanderson94/CIRCA_Clustering)*, using a Late 2015 iMac (macOS Catalina, 3.2 GHz Quad-Core Intel Core i5, 24 GB RAM). Figure S1 presents this data in terms of runtime (A-C) and number of coordinate ascent proposals (D-F).

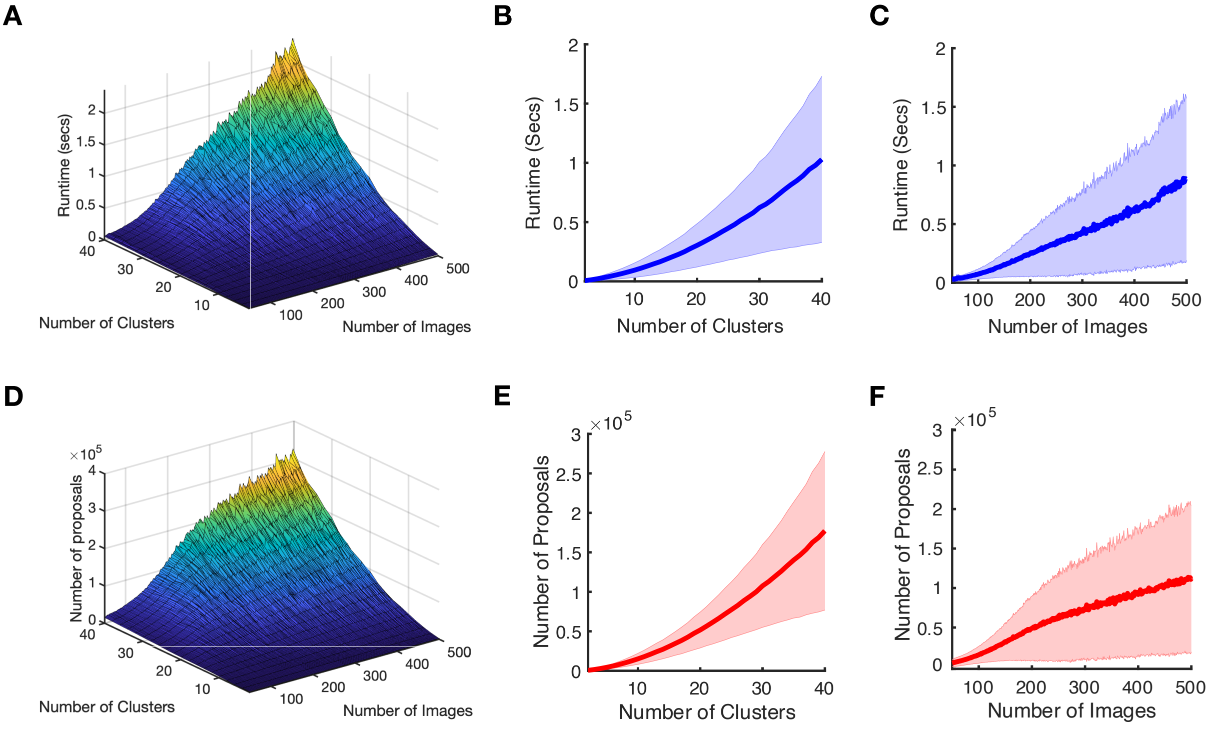

Figure S1. We empirically tested the time complexity of the CIRCA method by simulating noisy categorization data, and varying the number of images, n, and clusters, k. (A) Three-dimensional surface plot showing runtime (in seconds) as a function of n and k. (B) Runtime as a function of the k, averaging across n. This non-linear relationship is well described by a power law (with an exponent of 1.69). Shaded error bars give ± 1 standard deviation, and increases with k, indicating that optimization is more sensitive to the initialized clustering as k increases (likewise with n in C). (C) Runtime increases linearly as a function of the n, collapsing over k. (D) Number of proposals (i.e., accepted and rejected image reassignments)

made by the CIRCA method, as a function of n and k). (E) Collapsing over n reveals a non-linear relationship between k and number of moves, also described by a power law (with an exponent of 1.82). (F) Collapsing over k shows an approximately linear relationship.

Figure S1 demonstrates that runtime increases with n, following a power law, and linearly as a function of k (A-C). A highly similar pattern of growth is observed in the number of proposals (D-F). Moreover, the correlation between runtime and number of proposals is r =.99, indicating that the time complexity of the CIRCA method is primarily explained by the number of proposals required to reach convergence, i.e., by the size/complexity of the search-space. Next, we train a multiple regression to predict runtime from n and k, and examine the estimated runtime using much larger datasets. For results, see Table S1.

Table S1.

Estimated runtime for the CIRCA algorithm with a large number of images, and a variable number of clusters.

Number of Images	Number of Clusters	Est. Runtime (secs)
5000	5	1.79
50,000	5	48.46
5000	50	84.77
50,000	50	2293.57
5000	500	4011.92
50,000	500	108541.92

Supplementary Materials 2

To compare our coordinate ascent algorithm against competing clustering methods, we simulated data from two noisy participants. In this simulation, subjects 1 and 2 view 200 images, and respond using the same category system. Yet, on 50% of trials, they both select a random category. Note that the random noise is independent between subjects. For each clustering algorithm, we derive the clusters using the data from participant 1, and compare these clusters against the data from participant 2 by measuring the ARI. We simulate 100 datasets with k = 2:50 clusters/categories.

We selected two well-established algorithms for comparison: k-medoids, and spectral clustering (using default parameter settings; both algorithms were implemented using core MATLAB R2019B functions). A performant clustering method should be robust against response noise, and should generate above-chance predictions for test data (in our case, quantified by maximizing the ARI). Results are plotted in Figure S2.

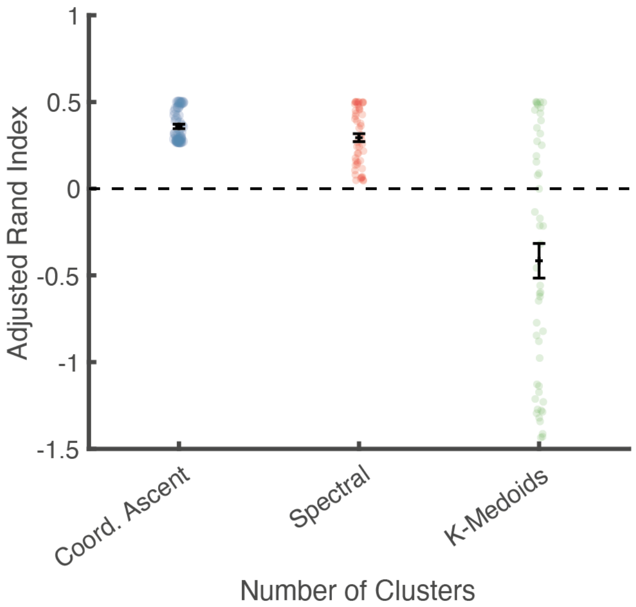

Figure S2. Our coordinate ascent method outperforms spectral and k-medoids clustering methods on simulated data from two hypothetical subjects. Individual datapoints represent the mean ARI per number of clusters (varying from 2-50). Error bars represent $\pm$ 1 standard error. Chance-performance is represented by the horizontal dashed line.

We observed that our coordinate ascent method produced a higher average ARI, and lower variance, compared to the two other algorithms. These results reveal that our method is more robust to response noise than other competing methods.

An additional requirement for our clustering algorithm is that is produces the 'correct' number of clusters when the number of clusters is known. Often, with experimental data, the number of clusters/categories is unknown, and we can only estimate it via procedures like cross-validation. By contrast, simulations offer complete control over the data. As above, we simulate the responses of two subjects. Subject 1 is an ideal observer that responds using the same category system on every trial. Subject 2 varies in the category system they use, and *disagrees* with participant 1 on a variable proportion of trials. When disagreement is 0, both subjects respond identically, and when disagreement is 1, subject 2 disagrees with subject 1 on every trial. Note that this is *not* the same as subject 2 responding randomly on some trials because, while subject 2 using a different category system to subject 1, all subject 2's responses are completely consistent with each other (put simply, each image can only belong to one category). Both subjects viewed 200 images, and used exactly 20 categories.

To test our method, we used 10-fold cross-validation on trial-by-trial data. We derived the optimal clustering for 90% of the dataset, and tested this clustering against the left-out 10% by measuring the ARI. We are primarily interested in testing the change in ARI as a function of the number of clusters used in the CIRCA method. When disagreement is modest, and both simulated subjects responded using more-or-less the same 20 categories, we should observe a maximum ARI at 20 clusters. Moreover, when disagreement is 0, we should perfectly reproduce the category system used by both subjects (ARI=1). However, when disagreement is high, it is unclear whether our method will overestimate, underestimate, or correctly estimate the number of clusters. Figure S3 plots ARI (between clusters fitted to 90% of the data, and the remaining 10% of the data) as a function of the number of clusters, and the proportion of disagreement. We also test the results produced by combining subject 1's responses with a completely random subject (black dashed line).

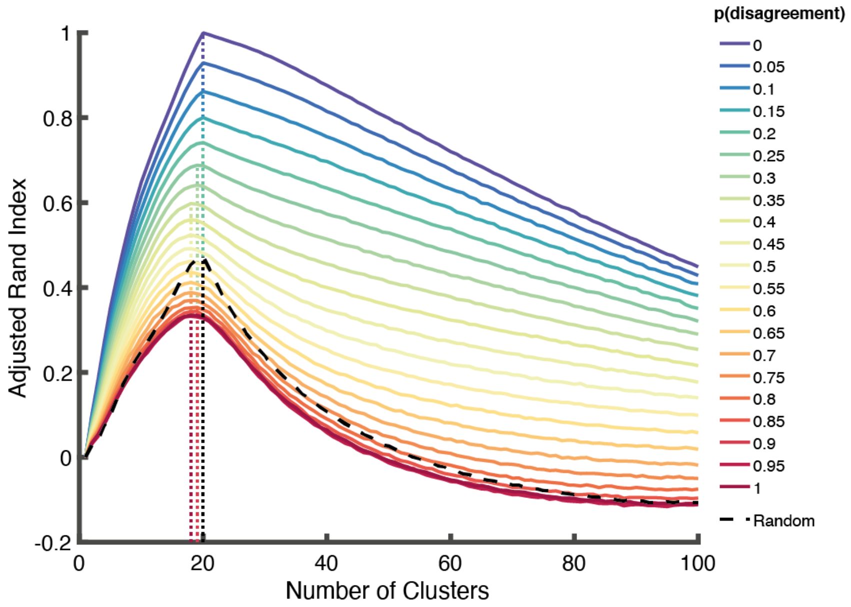

Figure S3. Our coordinate ascent method is robust against inter-subject disagreement and response noise. Individual solid lines show the average change in ARI as a function of the number of clusters, and different colours show how these trends vary with inter-subject disagreement (from blue to red). We also plot results from noisy data, where 50% of responses are random (black dashed line). For each level of disagreement, the maximum is identified with a dotted vertical line. Note that for each level of disagreement, we observe a single maximum at around 20 clusters, and see only a modest underestimation when inter-subject disagreement is high.

For moderate levels of disagreement (0-.25) we found that our method generated the correct number of clusters (vertical dotted lines). For greater levels of disagreement, our method underestimated the number of clusters, but only by 1-2. As expected, when disagreement was zero, our method perfectly reproduced the category system used by observers (ARI=1). Moreover, when we combined the responses from subject 1 with a subject that responded randomly on 100% of trials, we still observed a maximum at 20 clusters.

Given that few studies would predict total independence of subject responses, which would prohibit the generalization of results beyond the single subject, the modest underestimation in the number of clusters when subjects disagree on up to 100% of trials suggests that our method is highly robust against subject disagreement. Similarly, these

results demonstrate that our method produces reasonable results in the presence of large amounts of response noise (50% noise in this simulation).
